# USP44 inactivation accelerates the progression of thyroid cancer by inducing ubiquitylation and degradation of p21

**DOI:** 10.7150/ijbs.99817

**Published:** 2024-09-23

**Authors:** Yan Liu, Mengmeng Yuan, Xinxin Xu, Huini Yang, Yao Yao, Peng Hou, Wei Yu, Meiju Ji

**Affiliations:** 1 International Joint Research Center for Tumor Precision Medicine of Shaanxi Province and Department of Endocrinology, The First Affiliated Hospital of Xi'an Jiaotong University, Xi'an 710061, P.R. China.; 2 BioBank, The First Affiliated Hospital of Xi'an Jiaotong University, Xi'an 710061, P.R. China.; 3 Center for Translational Medicine, The First Affiliated Hospital of Xi'an Jiaotong University, Xi'an 710061, P.R. China.

**Keywords:** thyroid cancer, USP44, p21, deubiquitylation, cell cycle

## Abstract

Ubiquitin-specific peptidase 44 (USP44) belongs to the ubiquitin-specific protease family and is pivotal in the development and progression of tumors across various human cancers. However, its biological function and the underlying mechanisms in thyroid cancer remain poorly understood. In this study, we observed that *USP44* was frequently downregulated by promoter hypermethylation in thyroid cancers and found that its decreased expression was closely associated with poor patient survival. Subsequent *in vitro* and *in vivo* functional studies revealed that USP44 substantially suppressed the proliferation of thyroid cancer cells by impeding the G1/S transition in cell cycle. Mechanistically, USP44 directly interacted with p21 and eliminated its K-48-linked polyubiquitination chain, thereby stabilizing p21 proteins in a cell cycle-independent manner. In addition, the rescue of p21 partially alleviated cell cycle advancement and cell proliferation induced by the depletion of USP44. Our findings, taken together, indicate that USP44 is frequently repressed in thyroid cancer due to promoter hypermethylation and functions as a tumor suppressor by stabilizing p21 via deubiquitination.

## Introduction

Thyroid cancer stands as the preeminent malignancy within endocrine system 1, witnessing a notable surge in its incidence over recent epochs, notably among the female populace 2, 3. In general, thyroid cancer is histologically categorized into several types: papillary thyroid cancer (PTC), follicular thyroid cancer (FTC), medullary thyroid cancer (MTC), poorly differentiated thyroid cancer (PDTC) and anaplastic thyroid cancer (ATC). Predominantly, well-differentiated thyroid cancers (WDTCs), encompassing PTC and FTC, are renowned for their favorable prognosis. However, some patients may develop more aggressive forms of the condition, such as metastatic differentiated thyroid cancer (DTC), PDTC and ATC. These variants frequently exhibit resistance to standard treatments, leading to a grim prognosis 4. Hence, a compelling imperative arises to delve into the molecular underpinnings governing the de-differentiation and pathogenesis of thyroid cancer.

Ubiquitination is a dynamic and reversible phenomenon controlled by ubiquitylating and deubiquitylating enzymes (DUBs), which respectively add and remove ubiquitin signals 5. Among these enzymes, ubiquitin-specific protease 44 (USP44), a member of the ubiquitin-specific protease (USP) family, assumes the role of a deubiquitinating agent. Positioned on human chromosome 12(12q22), USP44 comprises a conserved USP domain along with a UBP-type zinc-finger (ZNF-UBP) domain 6, 7. Recent studies have shown that USP44 plays important roles in a plethora of physiological and pathological processes. For instance, USP44 functions as a tumor suppressor by regulating centrosome separation, localization and spindle geometry during mitosis to prevent the formation of aneuploidy 8. Moreover, USP44 has the ability to obstruct the assembly of downstream repair factors for DNA damage by impeding the polyubiquitination of histone H2A which is RNF168-mediated 9. USP44 has the capacity to regulate DNA double-strand break repair through TRIM25-Ku80 axis in nasopharyngeal carcinoma 10. Besides, USP44 is able to reverse H2B-K120 mono-ubiquitination mediated by RNF20, thereby regulating the normal differentiation of embryonic stem cells 11. Importantly, dysregulated expression of USP44 has been observed in numerous malignancies, with promoter methylation has been identified as an epigenetic regulatory mechanism controlling USP44 expression 10, 12, 13. However, the underlying roles of USP44 in thyroid cancer remain elusive.

The cyclin-dependent kinase (CDK) inhibitor p21, alternatively referred to as p21CIP1/WAF1 and coded by *CDKN1A*, acts as a crucial suppressor of cell-cycle advancement. It induces cell cycle arrest at the G1/S transition by blocking cyclin E and cyclin A/CDK2 activity through its amino-terminal CDK-cyclin inhibitory domain 14-16. In addition, through its carboxy-terminal PCNA-binding domain, p21 forms associations with proliferating cell nuclear antigen (PCNA), competitively displacing DNA polymerase-δ and thereby impeding DNA synthesis 15, 16. A series of studies have shown that p21 levels are regulated by both transcriptional and posttranslational mechanisms 17, 18 and its transcription is regulated in both p53-dependent and independent manners 17, 19. The ubiquitin-proteasome system is a predominant mechanism for controlling p21 levels via its degradation 20, and several E3 ubiquitin ligase complexes, including CRL4cdt2, SCFskp2 and APC/Ccdc20, trigger ubiquitylation and degradation of p21 in different stage of the cell cycle 21-23. However, p21 can be also stabilized by the interacting proteins that can reverse the ubiquitin-dependent degradation 24, 25.

In this study, we find that USP44 is downregulated by promoter hypermethylation in thyroid cancer and its downregulation is strongly correlated with unfavorable patient survival outcomes. Functional studies demonstrate that USP44 acts as a tumor suppressor in thyroid cancer cells. Further studies reveal that USP44 inhibits cell cycle progression by stabilizing p21 proteins via deubiquitination.

## Materials and Methods

### Clinical samples

Thirteen pairs of primary PTC tissues and corresponding adjacent noncancerous tissues (control subjects) were obtained from The First Affiliated Hospital of Xi'an Jiaotong University. Following acquisition, the specimens underwent fixation in 10% formaldehyde, paraffin embedding, and subsequent preparation of paraffin sections for immunohistochemical staining. All enrolled patients did not receive any therapeutic interventions and provided informed consent prior to surgical procedures. Histological examination of all tissues was conducted by two independent pathologists. This study received approval from the human ethics committee and the institutional review board.

### RNA extraction and quantitative Reverse Transcription PCR (qRT-PCR) assay

The detailed protocols were described in a previous study 26. Briefly, Trizol was added to the cells, followed by chloroform to separate the DNA and protein, leaving the RNA in the water phase. Concentrated RNA was precipitated by isopropyl alcohol and washed with 75% ethanol. The concentration and purity of RNA samples were measured by ultraviolet-spectrophotometer. Relative mRNA expression levels of the indicated genes were normalized to *β-actin* as an internal control, with each sample analyzed in triplicate. The primer sequences were presented in Table S1.

### DNA isolation and methylation-specific PCR (MSP)

Genomic DNA extraction, bisulfite modification and methylation-specific PCR (MSP-PCR) were conducted as described previously 26, 27. The primer sequences were presented in Table S1.

### Cell culture and drug treatment

Human thyroid cancer cell lines 8505C, 8305C, IHH4, SW1736, FTC133, BCPAP and K1, along with HTori-3 cell line derived from normal thyroid epithelial cells, were generously provided by Dr. Haixia Guan (Guangdong Provincial People's Hospital, Guangzhou, P.R. China). C643 cell line was obtained from Dr. Lei Ye (Ruijin Hospital, Shanghai, P.R. China). These cells were maintained consistently at 37 °C with 5% CO2 in RPMI 1640 or DMEM medium supplemented with 10% fetal bovine serum (FBS). In certain experiments, cells were treated with 5 μM 5-aza-20-deoxycytidine (5-Aza-dC; Sigma-Aldrich; 189825) to inhibit DNA methyltransferase (DNMT) activity for 5 days. Some cell lines were subjected to treatment with 20 μM proteasome inhibitor MG132 (Selleck Chemicals; S2619) or lysosome inhibitor 50 μM CQ (MCE; HY-17589A) for 6 h. To block newly protein synthesis, cells were treated with 200 μg/mL cycloheximide (CHX, Selleck Chemicals; S7418) for the indicated time points.

### Plasmids, siRNA and lentivirus transduction

The overexpression plasmid pCDNA3.1-USP44-Flag, pCDNA3.1-p21-Myc, USP44 deletion mutants, p21 truncation mutants, pCMV-HA-Ub and the ubiquitin mutants were all purchased from MiaoLing Plasmid (Wuhan, China). Cells at 75% confluence were transfected the plasmid using X-treme GENE HP DNA Transfection Reagent (Roche; 6366236001) pGEX-4T-1-GST-USP44 and pET-28a-His-p21 were purchased from Zoonbio Biotechnology (Nanjing, China).

Control siRNA (si-NC) and siRNAs specifically targeting USP44 (si-USP44#1 and si-USP44#2) and CDKN1A (si-CDKN1A) were obtained from Sangon Biotech (Sangon, Shanghai, China). Cells were transfected at 45-50% confluence using X-tremeGENE (Roche;4476093001). The sequences were presented in Table S2. The lentivirus encoding USP44 and control lentivirus, as well as lentivirus expressing shRNA targeting USP44 (sh-USP#1 and sh-USP#2) and control lentivirus (sh-NC) were purchased from GeneChem Co., Ltd. (Shanghai. China). The sequences were also presented in Table S2.

### Cell proliferation and colony formation assays

The detailed protocols were carried out as previously described 28. Briefly, 800 cells per well were seeded into a 96-well plate, with five replicate wells for each group. After cell adhesion, 20 µL of MTT was added to each well and incubated at 37°C for 4 h. The culture medium was then discarded, and 150 µL of DMSO was added to each well to dissolve the formazan crystals. The absorbance of each well was measured using a microplate reader. For colony formation assay, 1000 cells per well were seeded into a 6-well plate and cultured for 10 to 14 days. The surviving cell colonies were then fixed with methanol and stained with 0.5% crystal violet. Finally, the photographs were taken, and colonies were then counted. Each experiment was performed in triplicate.

### Cell cycle analysis

Cells were collected and fixed with 70% cold ethanol for at least 24 h. After fixation, they were centrifuged to separate the cells and washed with PBS buffer. Cells were then stained with propidium iodide (in PBS buffer) and finally analyzed by flow cytometry.

### Western blotting analysis

The protocol followed was consistent with a previous study 29. Briefly, cells were lysed using RIPA buffer, and denatured protein lysates were separated by SDS-PAGE. The proteins were then transferred onto a PVDF membrane. After blocking with BSA solution, the membrane was incubated overnight with the primary antibody listed in Table S3. The following day, the membrane was incubated with a horseradish peroxidase-conjugated secondary antibody (ZSGB-BIO), and immunoblot signals were visualized using an ECL detection kit (Millipore).

### Co-immunoprecipitation (Co-IP)

The cell lysates were subjected to overnight incubation at 4°C with primary antibodies or IgG, followed by a 4h incubation at 4°C with Protein A/G-agarose beads (Santa Cruz Biotechnology; sc2003). Immunoprecipitates were subsequently washed with RIPA buffer and detected by western blotting analysis.

### GST pull-down assay

The bacterially expressed recombinant human GST-USP44, and His-p21 were purchased from Zoonbio Biotechnology (Nanjing, China). The proteins were mixed with binding buffer and incubated with Glutathione-Sepharose beads (Beyotime, China; P2262) at 4 °C overnight. Following this, the beads underwent three washes, and the proteins bound to them were eluted using 50 μL of 20 mM GSH in Tris-HCl (pH 8.0), 150 mM NaCl. Immobilized protein complexes were then boiled and denatured at 95°C for western blotting analysis.

### Cell synchronization

Cell synchronization was conducted through the double thymidine block and release. Briefly, cells were treated with 2 mM thymidine (Sigma; T1895)) for 18 h, and released from the block by replacing normal medium for 9 h. Next, thymidine was reintroduced into the medium with a concentration of 2 mM for 18 h. Following this, cells were washed twice using PBS to arrest them in the G0/G1 phase and subsequently cultured in normal medium for 3 h (S phase cells) or 6 h (G2/M phase cells).

### EdU incorporation assay

The EdU incorporation assay was applied using an EdU Kit (Beyotime, China; C0071S). Cells from 6-well plates were treated with EdU reagent (diluted 1:1000) for 2.5 h. After that, cells were fixed with 4% paraformaldehyde, permeabilized with 1% Triton X-100, and stained with fluorescent dye. The proportion of EdU-positive cells was then analyzed by flow cytometry.

### Animal studies

All animal experiments were conducted with approval from the Laboratory Animal Center of Xi'an Jiaotong University. The female nude mice were obtained from GemPharmatech and kept in a specific pathogen-free (SPF) environment. To establish xenograft tumor model, nude mice aged 4-5 weeks were segregated into four groups. Then, 6×10^6^ 8505C cells stably overexpressing/knocking down USP44 and their control cells were injected into the right axillae of the nude mice, respectively. Tumor size and mouse weight were measured every 3 days, and computed the tumor volume with the formula: Tumor volume = length × width^2^ × 0.5. After 20 days, the mice were sacrificed and xenograft tumors were excised and weighed.

*TPO-Cre* mice were kindly provided by Dr. Shioko Kimura (National Cancer Institute). *LSL-Braf ^V600E^* mice were generously provided by Dr. Martin McMahon (The University of California, San Francisco). *LSL-Usp44* mice were purchased from Cyagen (Cyagen Biosciences). A mouse orthotopic thyroid cancer model was established as previously described 30. We identified the genotyping by extracting genomic DNA from mouse-tails and found that *Braf^V600E^*-driven mice developed thyroid cancer at approximately 6-weeks age. The animal Vevo 1100 ultrasound imaging system was used to access the thyroid tumor burden.

### Immunohistochemical (IHC) analysis

Immunohistochemical staining of USP44, p21, and Ki-67 was carried out as described previously 29. In brief, isolated tissues were fixed in formalin and embedded in paraffin. Sections of 4 μm thickness were placed on glass slides, deparaffinized in xylene and rehydrated through a graded ethanol series. Antigen retrieval was achieved by immersing the slides in 10 mM sodium citrate and boiling for 30 min. The sections were blocked for 1 h and incubated overnight at 4°C with primary antibodies. The next day, the slides were incubated with secondary antibodies and subsequently treated with 3,3'-diaminobenzidine (DAB). The primary antibodies used were also presented in Table S3. The slides were counterstained with hematoxylin, dehydrated and mounted with coverslips. Images were acquired using a Leica microscope slide scanner, and quantitative analysis was performed using ImageJ software. Ki-67 expression was quantified as the percentage of positive cells, while USP44 and p21 expression were evaluated by calculating the average optical density (AOD), using the formula: AOD = integrated optical density (IOD) / area.

### Immunofluorescence (IF) staining

Immunofluorescence assays were performed to assess the co-localization of USP44 and p21 as previously described 31. Briefly, cells were seeded onto coverslips in 12-well plates and cultured to 40% confluence. Cells were fixed in 4.0% paraformaldehyde for 15 min, permeabilized with 0.3% Triton X-100 for 10 min and blocked with 5% goat serum for 30 min. After blocking, cells were incubated overnight at 4°C with primary antibodies. The following day, cells were incubated at room temperature for 1.5 h with Alexa Fluor-conjugated goat anti-rabbit or goat anti-mouse secondary antibodies (Invitrogen). Finally, nuclei were stained with Hoechst 33342, and confocal images were obtained using a laser scanning microscope (Leica, Wetzlar, Germany).

### Statistical analysis

All data were analyzed using GraphPad Prism 8.3.0 software. Statistical significance between two groups was determined using Student's t-test, while one-way ANOVA was used for multiple group comparisons. The Spearman's correlation test was performed in correlation analyses. Each experiment was conducted at least 3 times and the data were presented as mean ± standard deviation (SD). *P* < 0.05 indicated statistical significance.

## Results

### USP44 is downregulated by promoter hypermethylation in thyroid cancer

**Figure 1:**
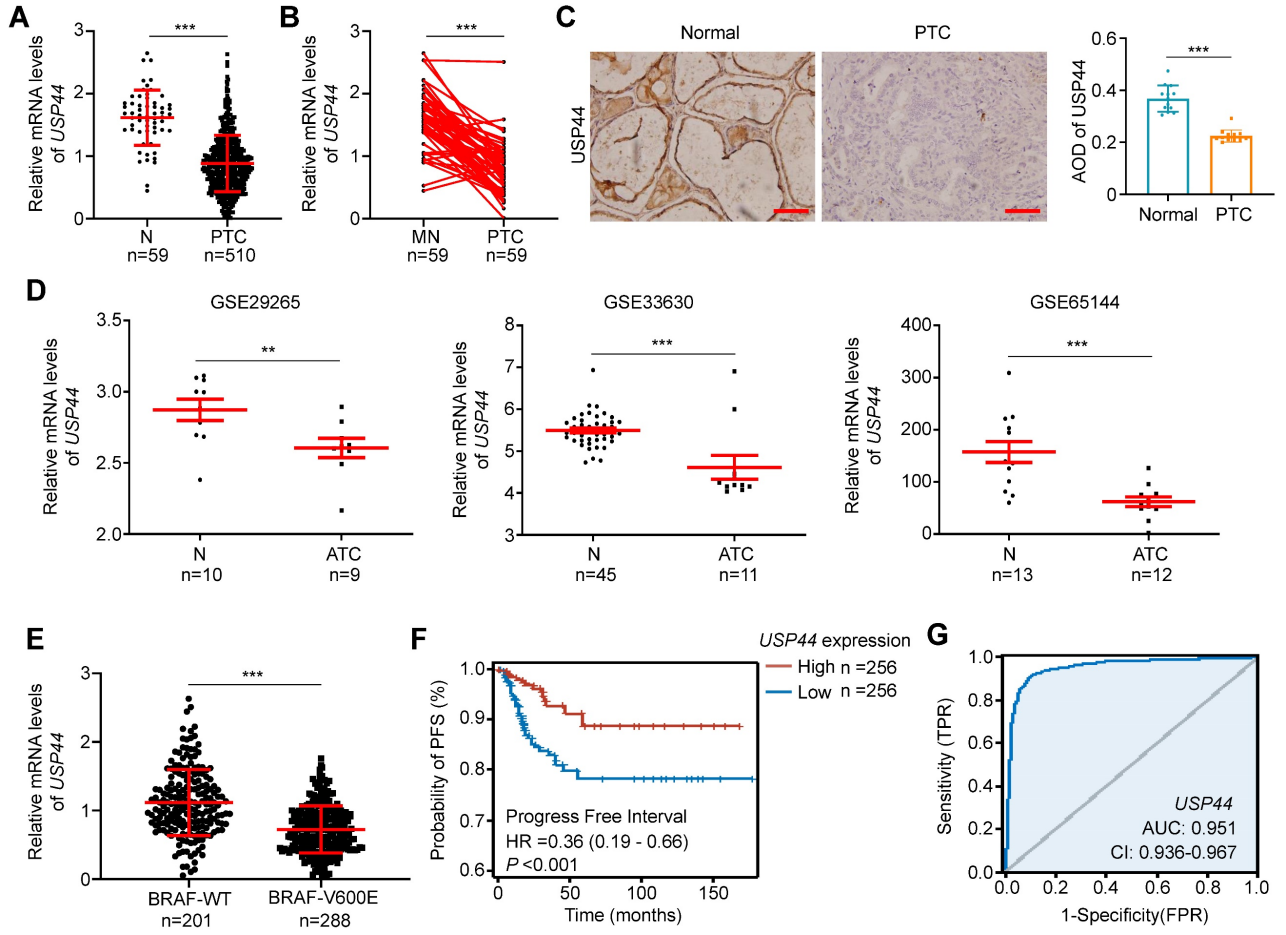
**USP44 downregulation and its clinical significance in thyroid cancer. A** Analysis of *USP44* mRNA expression in papillary thyroid carcinoma (PTC) and normal thyroid tissues (N) in The Cancer Genome Atlas (TCGA) dataset. **B** Comparison of *USP44* mRNA expression in PTC and matched noncancerous tissues (MN) in the TCGA dataset. **C** Immunohistochemical staining images of USP44 in PTCs and matched noncancerous tissues (Normal). Representative images were shown in the left panel, with corresponding statistical plots presented in the right panel. Scale bar, 50 μm. **D** Analysis of *USP44* mRNA expression levels in anaplastic thyroid carcinoma (ATC) and noncancerous tissues (N) based on GEO database. **E** Analysis of *USP44* mRNA expression in PTC with different *BRAF* mutation statuses. **F** Progression-free interval (PFI) from PTC dataset with low or high *USP44* expression. **G** Diagnostic ROC curve of *USP44*. The data were expressed as the mean ± SD. **, *P* < 0.01; ***, *P* < 0.001; ns, no significance.

Using the RNA-Seq dataset from The Cancer Genome Atlas (TCGA) database, we investigated mRNA expression of *USP44* in PTC tissues and non-cancerous thyroid tissues. The results showed that *USP44* expression in PTC tissues was remarkably lower than that in non-cancerous control (Fig. 1A) or their matched normal thyroid tissues (Fig. 1B). We next examined protein expression of USP44 in 13 PTCs and their matched non-cancerous tissues by IHC staining and confirmed the decreased expression of USP44 in PTCs compared to control tissues (Fig. 1C). In addition, we also analyzed mRNA expression of *USP44* in ATCs and non-cancerous thyroid tissues using the Gene Expression Omnibus (GEO) datasets. Expectedly, *USP44* had a lower expression in ATC tissues than control tissues (Fig. 1D).

By further analyzing the PTC dataset from TCGA database, we observed that *USP44* expression exhibited a negative association with *BRAF^V600E^* mutation (Fig. 1E) and a positive relationship with progression-free interval (PFI) of patients (Fig. 1F). Notably, we generated the ROC curves of *USP44* using TCGA dataset, and found that its area under the curve (AUC) was 0.951 (Fig. 1G), suggesting its potential as a biomarker for the diagnosis of PTC. In addition, we also investigated the correlation between *USP44* expression and clinicopathologic characteristics of PTC patients using the TCGA database. As shown in Table S4, *USP44* expression was negatively associated with T stage (*P* < 0.001), N stage (*P* = 0.002), pathologic stage (*P* < 0.001), age (*P* = 0.027), histological type (*P* < 0.001), extrathyroidal extension (*P* < 0.001) and thyroid gland disorder history (*P* = 0.008).

**Figure 2:**
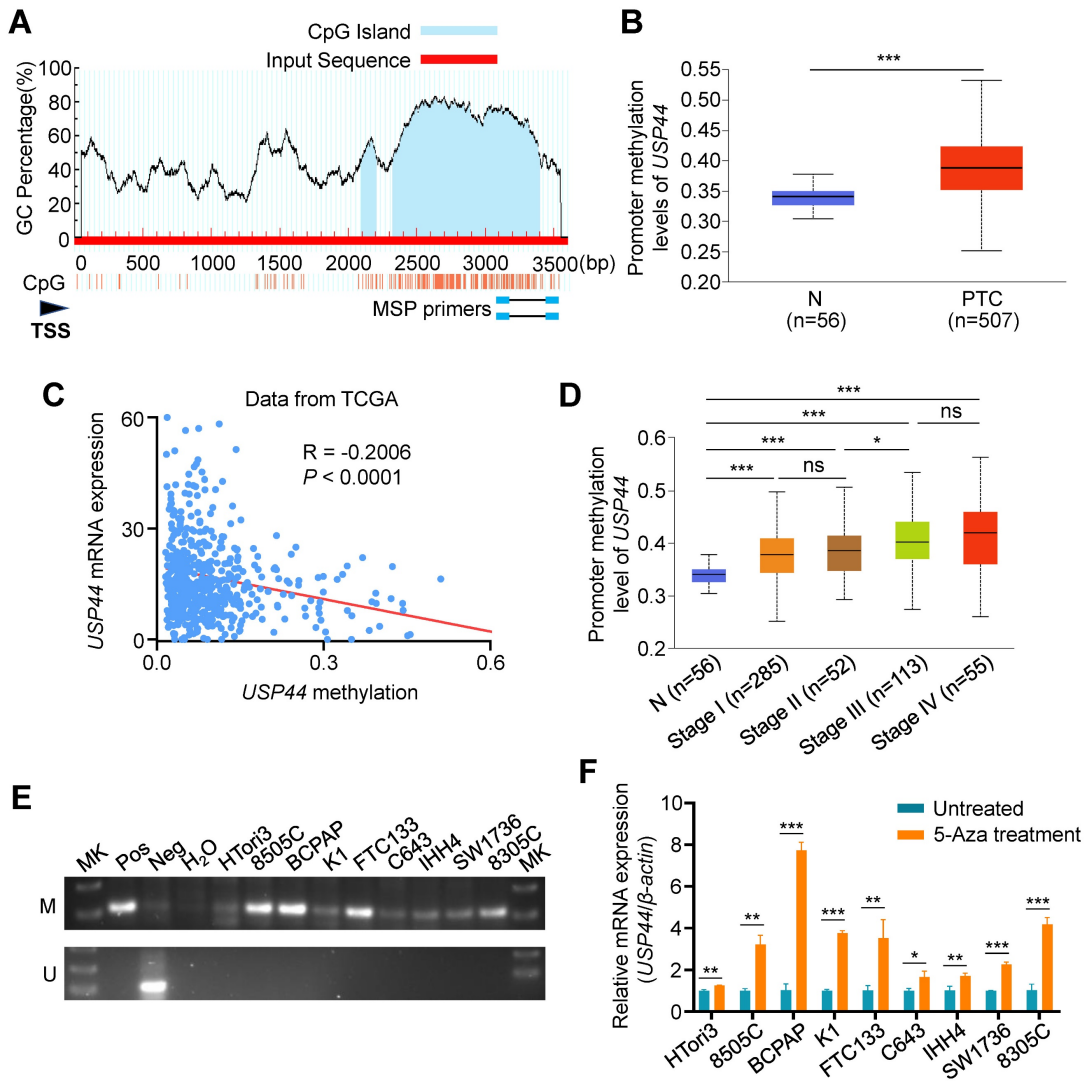
**Promoter hypermethylation-mediated *USP44* downregulation in thyroid cancer. A** Schematic illustration depicting the promoter region of USP44 analyzed for methylation, with CpG sites denoted by blue vertical lines. TSS, transcription start site. **B** The methylation levels of *USP44* promoter in PTCs and normal tissues (N) using UALCAN. **C** Association between methylation levels of *USP4*4 promoter and its mRNA levels (data from TCGA database). **D** Correlation of *USP44* methylation and pathological clinical stage in PTC (data from UALCAN database). **E** Methylation status of *USP44* promoter in thyroid cancer cells assessed by MSP assay. Bisulfite-modified normal leukocyte DNA was used as positive control for unmethylated gene (Neg); *in vitro* methylated DNA served as positive control for methylated gene (Pos); Mk, DNA marker; M represents amplified product using primers specific to the methylated sequence; U represents amplified product using primers specific to the unmethylated sequence. **F** mRNA expression of *USP44* in thyroid cancer cell lines upon 5-AZA-deoxycitidine treatment. The data were expressed as the mean ± SD from three independent experiments. *, *P* < 0.05; **, *P* < 0.01; ***, *P* < 0.001; ns, no significance.

It is the fact that promoter hypermethylation acts as a prominent inactivation mechanism of tumor suppressor genes in tumorigenesis, including thyroid cancer 32-34. Therefore, we utilized the Meth-Primer database to predict and validate the existence of CpG island within the promoter region of *USP44* (Fig. 2A). Next, we compared the methylation levels of *USP44* promoter between PTCs and non-cancerous thyroid tissues using UALCAN tool, revealing a significant increase in the levels of *USP44* methylation in PTCs compared to control tissues (Fig. 2B). Further analysis found that *USP44* methylation had a negative relationship with its mRNA levels (R = -0.2006, *P*<0.0001) (Fig. 2C) and a positive association with pathologic stage (Fig. 2D), supporting the above conclusion. Meanwhile, we analyzed *USP44* mRNA expression and methylation levels, and investigated their relationship in other 10 tumor types using the TCGA database. The results were consistent with those observed in thyroid cancer (Fig. S1A-C).

We next conducted MSP assay to detect *USP44* methylation in a panel of thyroid cancer cell lines and immortalized thyroid follicular cell line HTori3, and found different levels of *USP44* methylation in each of these thyroid cancer cell lines compared to HTori3 cell line (Fig. 2E). To ascertain whether *USP44* is inactivated by promoter methylation, we treated these cell lines with DNMT inhibitor 5-Aza-dC, and found that *USP44* expression was significantly restored in these cell lines upon 5-Aza-dC treatment (Fig. 2F). Collectively, our data indicate that promoter methylation of *USP44* is one of the major mechanisms of its downregulation in thyroid cancer.

### USP44 inhibits thyroid cancer cell cycle progression and *in vitro* growth

**Figure 3:**
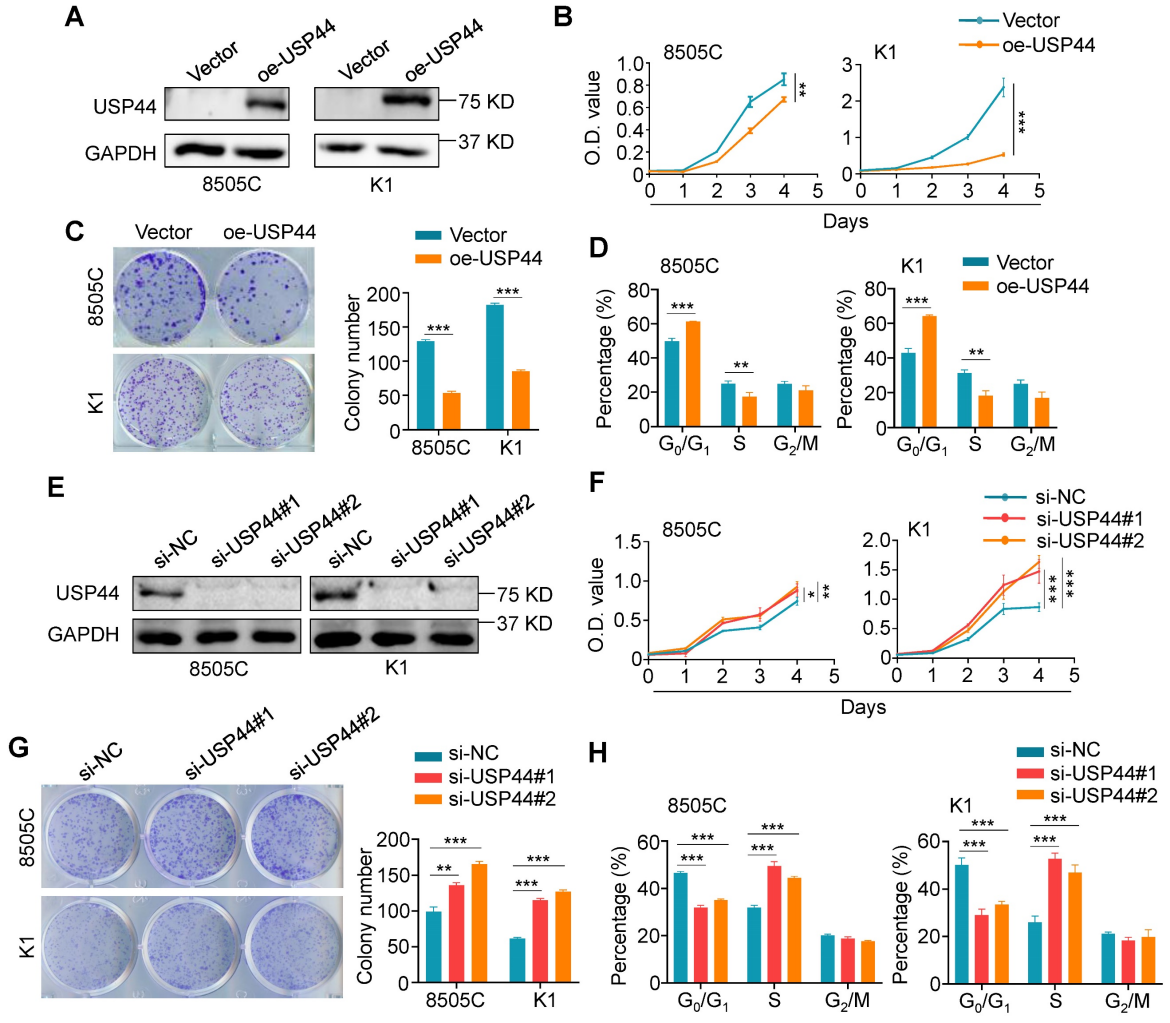
***In vitro* functional studies of USP44 in thyroid cancer cells. A** Western blotting analysis confirming ectopic expression of Flag-tagged USP44 in 8505C and K1 cells. **B** MTT assay was employed to detect the inhibitory effect of ectopic expression of USP44 in 8505C and K1 cells on cell proliferation. **C** The effect of ectopic expression of USP44 on colony formation ability in 8505C and K1 cells. Left panels show representative colony formation images and the right panel presents corresponding statistical analyses. **D** The effect of ectopic expression of USP44 on cell cycle distribution was analyzed by flow cytometry. **E** Efficient knockdown of USP44 in 8505C and K1 cells was validated by western blotting analysis, with GAPDH as a loading control. **F** The effect of knocking down USP44 in 8505C and K1 cells on cell proliferation was assessed by MTT assays. **G** The effect of knocking down USP44 in 8505C and K1 cells on colony formation ability. Left panels show representative colony formation images and the right panel presents the statistical results. **H** The effect of knocking down USP44 in 8505C and K1 cells on cell cycle progression was evaluated by flow cytometry. The data were presented as the means ± SD. *, *P* < 0.05; **,* P* < 0.01; ***, *P* < 0.001.

To determine the potential role of USP44 in thyroid cancer, we performed a series of *in vitro* functional experiments in thyroid cancer cells. We first evaluated the effect of ectopic expression of USP44 in 8505C and K1 cells (Fig. 3A) on their malignant phenotypes. The results showed that ectopic expression of USP44 evidently inhibited the proliferation and colony formation ability of thyroid cancer cells (Fig. 3B and C). Subsequently, flow cytometry was conducted to interrogate the cell cycle distribution, confirming the induction of cell cycle arrest at the G0/G1 phase specifically upon ectopic expression of USP44 (Fig. 3D). Conversely, knocking down USP44 in 8505C and K1 cells markedly augmented cell proliferation and colony formation abilities compared to control cells (Fig. 3E-G). Moreover, USP44 knockdown significantly facilitated the G1/S phase transition (Fig. 3H). Altogether, these findings strongly indicate that USP44 is essential for cell cycle regulation and acts as a tumor suppressor in thyroid cancer cells.

### USP44 suppresses thyroid cancer cell growth *in vivo*

**Figure 4:**
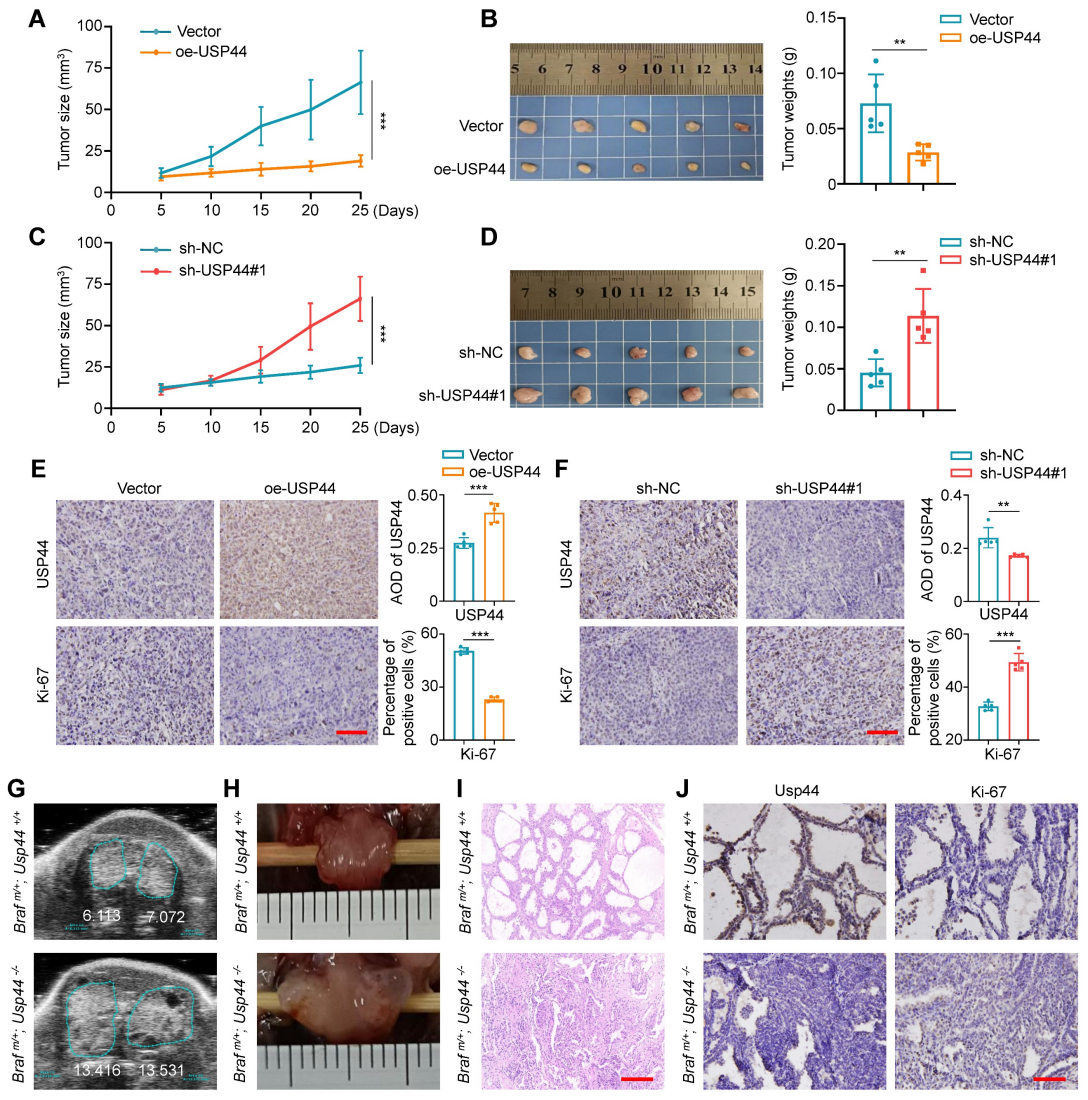
**Inhibitory effect of USP44 on tumor growth in xenograft and transgenic mouse models. A** Tumor growth curves comparing nude mice subcutaneously injected with USP44-overexpressing 8505C cells versus control cells (n = 5/group). Day 0 represents the time point of tumor cell injection. **B** Photographs of dissected tumors from nude mice (left panel). Right panel shows corresponding tumor weights from the two groups. **C** Growth curves of USP44-knockdown tumors and control tumors (n = 5/group). Day 0 represents the time point of tumor cell injection. **D** Photographs of dissected tumors from nude mice (left panel). Right panel shows corresponding tumor weights from the two groups. IHC staining of USP44 and Ki-67 in USP44-overexpression (**E**) and -knockdown (**F**) tumor tissues as well as their control tissues. Representative images were shown in the left panel, with corresponding statistical plots presented in the right panel. Representative images of ultrasound (**G**), gross appearance (**H**) and H&E staining (**I**) of thyroid tumors from transgenic mice with indicated genotypes. **J** IHC staining of Usp44 and Ki-67 in tumor tissues from the indicated mice. The data were presented as the means ± SD. **,* P* < 0.01; ***, *P* < 0.001. Scale bar, 50 μm.

We also assessed the tumor-suppressive effect of USP44 using xenograft tumor models. As shown in Fig. 4A and B, USP44-overexpressing 8505C cell-derived xenograft tumors showed significantly slower growth and reduced tumor burden in contrast to the control group. On the contrary, USP44-knockdown 8505C cell-derived xenograft tumors displayed accelerated growth, with a remarked increase in both tumor volume and weight (Fig. 4C and D). Next, we performed IHC assays to measure the levels of USP44 and Ki-67 in the above tumor tissues. As shown in Fig. 4E, USP44-overexpressing tumor tissues exhibited a higher expression of USP44, while the percentage of Ki-67-positive cells showed a marked decrease compared to control tumor. In contrast, the levels of USP44 exhibited a notable reduction in the USP44-knockdown tumors when compared to control tumors, accompanied by an elevated percentage of Ki-67‑positive cells (Fig. 4F).

To further determine whether USP44 deficiency exacerbates the progression of thyroid cancer, we generated thyroid-specific *Usp44* knockout mouse model of *Braf^V600E^*-induced thyroid cancer by crossing *LSL-Braf^V600E^*, *LSL-Usp44* and thyroid peroxidase* (Tpo)-Cre* mice. This allowed for the development of spontaneous thyroid cancer with various genotypes. The transgenic mice were verified through genotyping of the mouse tail DNA (Fig. S2A-C). Follow-up ultrasound scan showed that thyroid tumors in homozygous *Usp44* knockout mice (*Braf ^m/+^; Usp44^-/-^*) grew more rapidly than that in *Usp44* wild-type mice *(Braf ^m/+^; Usp44^+/+^)* at tenth week (Fig. 4G; Fig. S2D), consistent with the gross appearance following dissection (Fig. 4H). We also analyzed histological pathology of thyroid tumors at sixth week by hematoxylin and eosin (H&E), and found that *Usp44* knockout combined with *Braf^V600E^* accelerated the formation of papillary thyroid architecture compared to *Braf^V600E^* alone (Fig. 4I). Next, we performed IHC assays to assess the levels of Usp44 and Ki-67 in the above thyroid tumor tissues and found that *Braf ^m/+^*/*Usp44^-/-^* mice had lower levels of Usp44 but higher levels of Ki-67 than *Braf ^m/+^; Usp44^+/+^* mice (Fig. 4J; Fig. S2E). These data, taken together, indicate the thyroid-specific *Usp44* knockout accelerates the progression of *BRAF^V600E^*-induced thyroid cancer, further highlighting the role of USP44 as a tumor suppressor in thyroid cancer.

### USP44 physically interacts with p21

**Figure 5:**
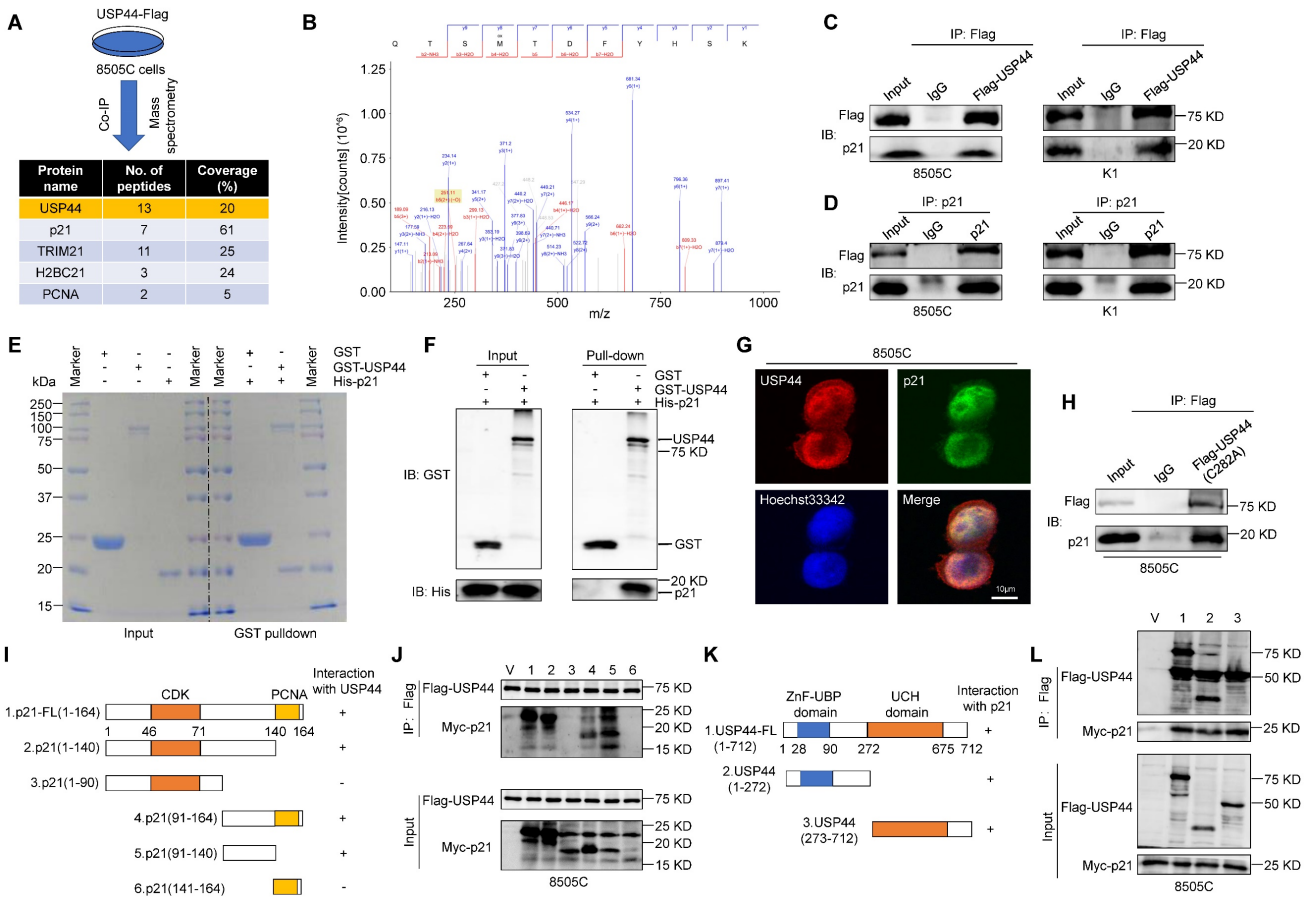
**Direct physical interaction between USP44 and p21. A** Schematic representation of Immunoprecipitation coupled with mass spectrometry (IP-MS) to identify proteins interacting with USP44. **B** p21 as USP44 interactor identified by LC-MS/MS. Cell lysates of 8505C and K1 cells expressing Flag-USP44 were subjected to immunoprecipitation using either anti-Flag (**C**) or anti-p21 antibody (**D**) followed by immunoblotting of the immunoprecipitates with anti-p21 or anti-Flag antibody. Immunoprecipitation using isotype IgG is negative control for co-IP. **E** The presence of recombinant GST, GST-USP44 and His-p21 proteins in the input samples and the proteins pulled down by GST were confirmed by SDS/PAGE and Coomassie blue staining.** F** The GST pulled down samples were detected using GST and His antibody. **G** Immunofluorescence assay was performed to determine the subcellular localization of endogenous USP44 (red) and p21 (green). Scale bar, 10 μm. **H** 8505C cells were transfected with catalytically inactive mutant of USP44 (C282A) and cell lysates underwent immunoprecipitation using anti-Flag antibody. **I** Schematic illustration depicting Myc-tagged full-length (FL) p21 and its various deletion mutants. **J** 8505C cells were transiently transfected with the specified constructs of p21 and Flag-tagged USP44. Cell lysates were then subjected to immunoprecipitation with anti-Flag antibody, and the immunoprecipitates were analyzed using anti-Myc and anti-Flag antibody. **K** Diagram illustrating the Flag-tagged full-length (FL) USP44 and its different deletion mutants. **L** 8505C cells were transiently transfected with the specified constructs of USP44 and Myc-tagged p21. Cell lysates then underwent immunoprecipitation using an anti-Flag antibody, and the resulting immunoprecipitates were analyzed with anti-Flag and anti-Myc antibodies.

As a deubiquitinating enzyme, USP44 is able to stabilize targeted proteins by cleaving ubiquitin-protein bonds. To explore the potential mechanism by which USP44 acts as a tumor suppressor in thyroid cancer, we performed co-immunoprecipitation (co-IP) assays followed by identification of co-precipitating proteins using liquid chromatography tandem mass spectrometry (LC-MS/MS), showing that p21 was detected in the Flag-tagged USP44 immunoprecipitates (Fig. 5A and B). Given that p21 serves as a cyclin-dependent kinase inhibitor and is regulated by ubiquitination degradation mechanisms, we speculate that USP44 may regulate cell cycle by interacting with p21.

To confirm the interaction between USP44 and p21, Flag-USP44 were transfected into 8505C and K1 cells, followed by reciprocal co-IP assays using either anti-Flag or anti-p21 antibody, demonstrating that Flag-USP44 interacted with endogenous p21 protein (Fig.5C and D). To further assess the direct interaction between USP44 and p21, we acquired purified recombinant GST-USP44 and His-p21 proteins, conducting GST pull-down assays. As shown in Fig. 5E and F, purified GST-USP44, rather than GST control, exhibited binding affinity towards His-p21, indicating that a direct interaction between USP44 and p21. Immunofluorescence assay further showed that the colocalization of USP44 and p21 was mainly in the nucleus (Fig. 5G).

To determine whether the enzyme catalytic activity of USP44 is critical for interacting with p21, we constructed a USP44 mutant (C282A) with impaired deubiquitinase activity, but maintained the binding ability with p21 (Fig. 5H). Furthermore, to identify the pivotal functional domain of p21 that interacted with USP44, we constructed a series of p21-truncated mutants (Fig. 5I). The results indicated that the C-terminal region (amino acids 91-140) of p21 could interact with USP44 (Fig. 5J). Conversely, we generated two deletion mutants containing either the C terminus ZnF-UBP domain or N terminus USP catalytic domain of USP44 (Fig. 5K), all tested USP44 domains were found to interact with p21 (Fig. 5L). Taken together, the above findings demonstrate that USP44 has direct physical interaction with p21.

### USP44 deubiquitinates and stabilizes p21

**Figure 6:**
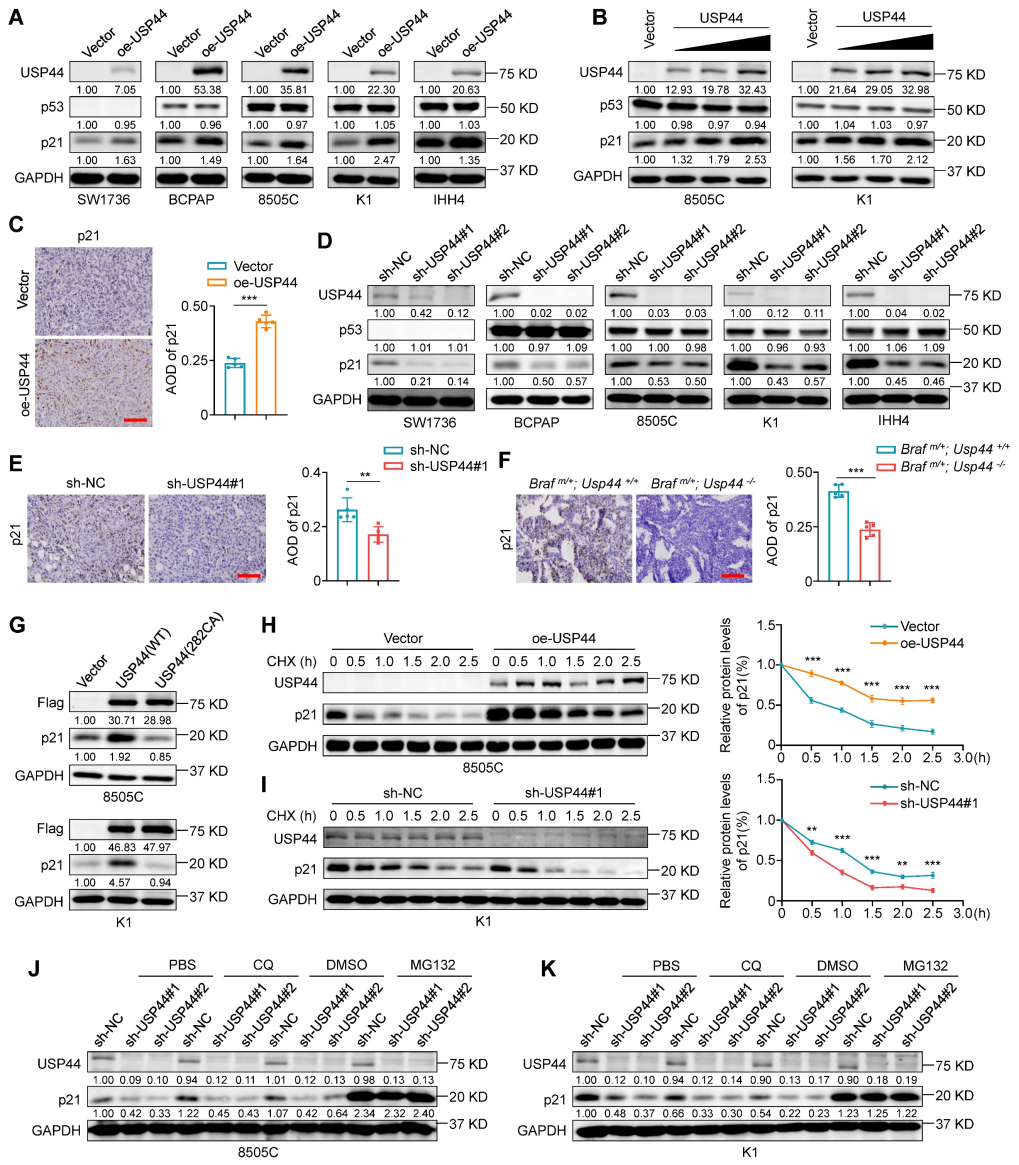
**USP44 increases the protein stability of p21. A** Western blotting demonstrating the effect of ectopic expression of Flag-tagged USP44 on the expression of p21 and p53 in the indicated thyroid cancer cells. **B** Increasing amounts of Flag-USP44 was transfected into 8505C and K1 cells, and these cell lysates underwent western blotting analysis to detect protein levels of p21 and p53. **C** IHC staining of p21 in USP44-overexpressing tumor tissues and control tissues. Representative images were shown in the left panel, with corresponding statistical plots presented in the right panel. Scale bar, 50 μm.** D** Western blotting analysis shows the effect of USP44 knockdown on the protein levels of p21 and p53 in the indicated thyroid cancer cells. **E** IHC staining of p21 in USP44-knockdown tumor tissues and control tissues. Representative images were shown in the left panel, with corresponding statistical plots presented in the right panel. Scale bar, 50 μm. **F** IHC staining of p21 in tumor tissues from transgenic mice with different genotypes. Representative images were shown in the left panel, with corresponding statistical plots presented in the right panel. Scale bar, 50 μm. **G** Western blotting analysis was utilized to detect protein levels of p21 in 8505C and K1 cells transfected with either wild-type USP44 (WT) or mutant USP44-C282A (C282A). USP44-overexpressing 8505C cells (**H**) and USP44-knockdown K1 cells (**I**) as well as their control cells were subjected to treatment with 200 μg/mL CHX and collected at the indicated time points for western blotting analysis (left panels). Quantification of p21 protein levels relative to GAPDH was shown in the right panels. USP44-knockdown 8505C cells (**J**) and K1 cells (**K**) were subjected to treatment with either 20 μM of MG132 or 50 μM of CQ for 6 h prior to harvesting and the resulting cell lysates were analyzed by western blotting analysis. Densitometric analysis of the specified proteins, normalized to GAPDH as a loading control and expressed as fold changes relative to the control group, was presented below the corresponding bands. The densitometric analysis of the indicated proteins, normalized to GAPDH as the loading control and expressed as fold change from control group, was displayed beneath the corresponding band. GAPDH was used as a loading control. The data were presented as the mean ± SD. **,* P* < 0.01; ***, *P* < 0.001.

We next investigate whether the interaction between USP44 and p21 affects the protein expression of the latter. Firstly, we utilized lentiviral transfection to ectopically express USP44 in a panel of thyroid cancer cell lines, including SW1736 (ATC, p53^-/-^), BCPAP (PTC, p53^mut^), 8505C (ATC, p53^mut^), K1 (PTC, p53^wt^) and IHH4 (ATC, p53^wt^). The results showed that the ectopic introduction of USP44 resulted in a substantial elevation of p21 protein expression in all cell lines (Fig. 6A), and demonstrated that this effect was dose-dependent, but not dependent on the status of p53 (Fig. 6A and B). Moreover, USP44 overexpression did not affect the protein levels of p53, indicating that USP44 regulates p21 expression in a p53-independent manner (Fig. 6B). Consistent with those *in vitro* observations, the USP44-overexpressing xenograft tumors exhibited a higher expression of p21 than control tumors (Fig. 6C). To further corroborate that USP44 regulates endogenous p21 levels, we conducted the loss-of-function experiments utilizing two lentiviral-based shRNAs to knockdown USP44 in the above mentioned 5 cell lines. As expected, the downregulation of USP44 dramatically decreased p21 levels without affecting p53 expression (Fig. 6D). Similarly, there was a notable reduction in p21 expression observed in USP44-knockdown xenograft tumors compared to control tumors (Fig. 6E). The orthotopic tumor models also demonstrated that tumor tissues from *Braf ^m/+^*/*Usp44^-/-^* mice had lower expression of p21 than that from *Braf ^m/+^*/*Usp44^+/+^* (Fig. 6F), further supporting the above conclusion. However, the catalytic inactive mutant USP44 (C282A) exhibited an inability to stabilize p21 expression (Fig. 6G), suggesting that USP44-mediated p21 upregulation may be dependent on its deubiquitinase activity.

To determine whether USP44 upregulates p21 at the transcriptional levels or post-transcriptional levels, p21 mRNA levels were analyzed by qRT-PCR. The results demonstrated that either USP44 overexpression or knockdown almost did not affect the levels of p21 mRNA (Fig. S3A and B), indicating that this regulation does not occur at transcriptional levels. Considering that USP44 can stabilize the targeted proteins via deubiquitination, we next explore whether USP44 can enhance the stability of p21 proteins. To this end, we treated USP44-overexpressing 8505C or USP44-knockdown K1 cells and their control cells with a protein synthesis inhibitor cycloheximide (CHX) for different time points to assess its effect on the protein levels of p21 by western blotting analysis. The results showed that, compared to the controls, USP44 overexpression significantly prolonged the half-life of p21 proteins (Fig.6H; Fig. S3C), while USP44 knockdown accelerated p21 degradation (Fig.6I; Fig. S3D). To further determine the pathway responsible for triggering p21 degradation, we treated USP44-knocking down cells and control cells with the lysosome inhibitor chloroquine (CQ) or proteasome inhibitor MG132 for 6 h. The results showed that the inhibitory effect of USP44 knockdown on p21 protein expression could be reversed by MG132 but not CQ (Fig. 6J and K). These data, taken together, indicate that USP44 stabilizes p21 proteins through suppressing its proteasome degradation.

**Figure 7:**
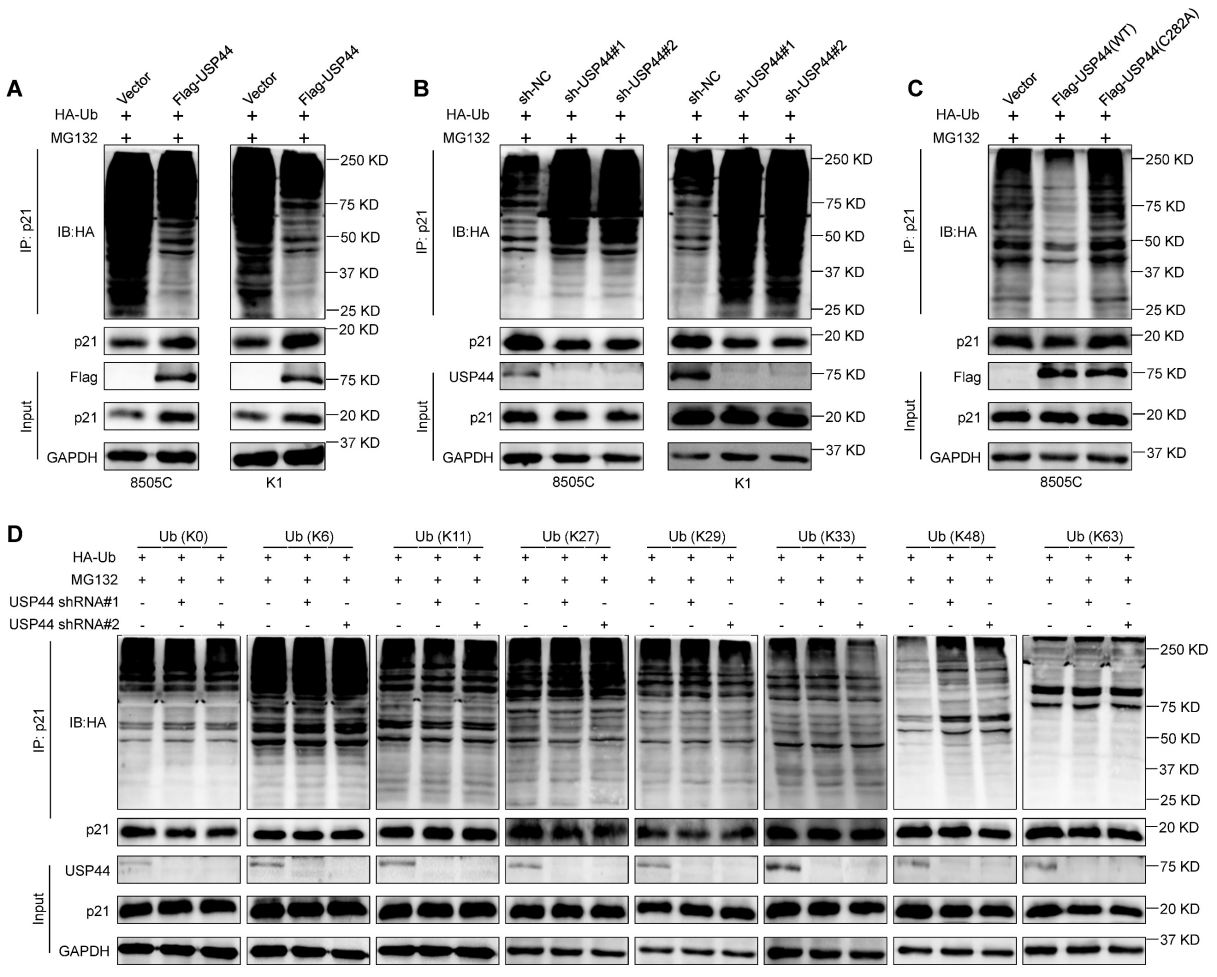
**USP44 deubiquitinates K48-linked chains on p21.** USP44-overexpressing 8505C and K1 cells (**A**), USP44-knockdown 8505C and K1 cells (**B**) and their control cells were treated with 20 μM MG132 for 6 h in the presence of HA-tagged ubiquitin (HA-Ub). The ubiquitination levels of p21was then assessed by western blotting analysis. **C** 8505C cells ectopically expressing either wild-type Flag-USP44 or Flag-USP44 mutant C282A and their control cells were treated with 20 μM MG132 for 6 h in the presence of HA-Ub. Western blotting analysis was then performed to evaluate their effect on ubiquitination levels of p21. **D** USP44-knockdown 8505C cells and control cells were transfected with various HA-tagged ubiquitin mutants and then treated with 20 μM MG132 for 6 h in the presence of HA-Ub. Next, western blotting analysis was performed to determine the effect of the above treatments on ubiquitination levels of p21.

To explore the underlying mechanism by which USP44 regulates p21 stability, we determined the effect of USP44 on the ubiquitination levels of p21 proteins by co-IP assays. As shown in Fig. 7A, the overexpression of Flag-USP44 decreased the polyubiquitination levels of p21 in 8505C and K1 cells, whereas USP44 knockdown elevated its polyubiquitylation levels (Fig. 7B). Furthermore, we transfected wild-type USP44 or USP44-C282A mutant together with HA-Ub into 8505C cells, showing that catalytically inactive mutant could not decrease p21 ubiquitination levels (Fig. 7C). This was consistent with the above findings that USP44-C282A mutant failed to stabilize p21, indicating that the enzymatic activity of USP44 is essential for the deubiquitination of p21. To ascertain the deubiquitination types of p21 by USP44, we transfected USP44-knockdown 8505C cells and control cells with different HA-tagged ubiquitin mutants. These mutants include the lysines mutant with all lysines mutated (K0) or lysines mutated into arginines except for the specific lysine site (K6, K11, K27, K29, K33, K48 or K63). As shown in Fig. 7D, USP44 knockdown increased the ubiquitination of p21 only in the presence of K48-Ub mutant but not any other isopeptide-linked poly-Ub (K6, K11, K27, K29, K33 or K63), indicating that the USP44 removes the K48-linked polyubiquitination chains in p21 proteins.

### USP44 regulates cell cycle progression in a p21-dependent manner

**Figure 8:**
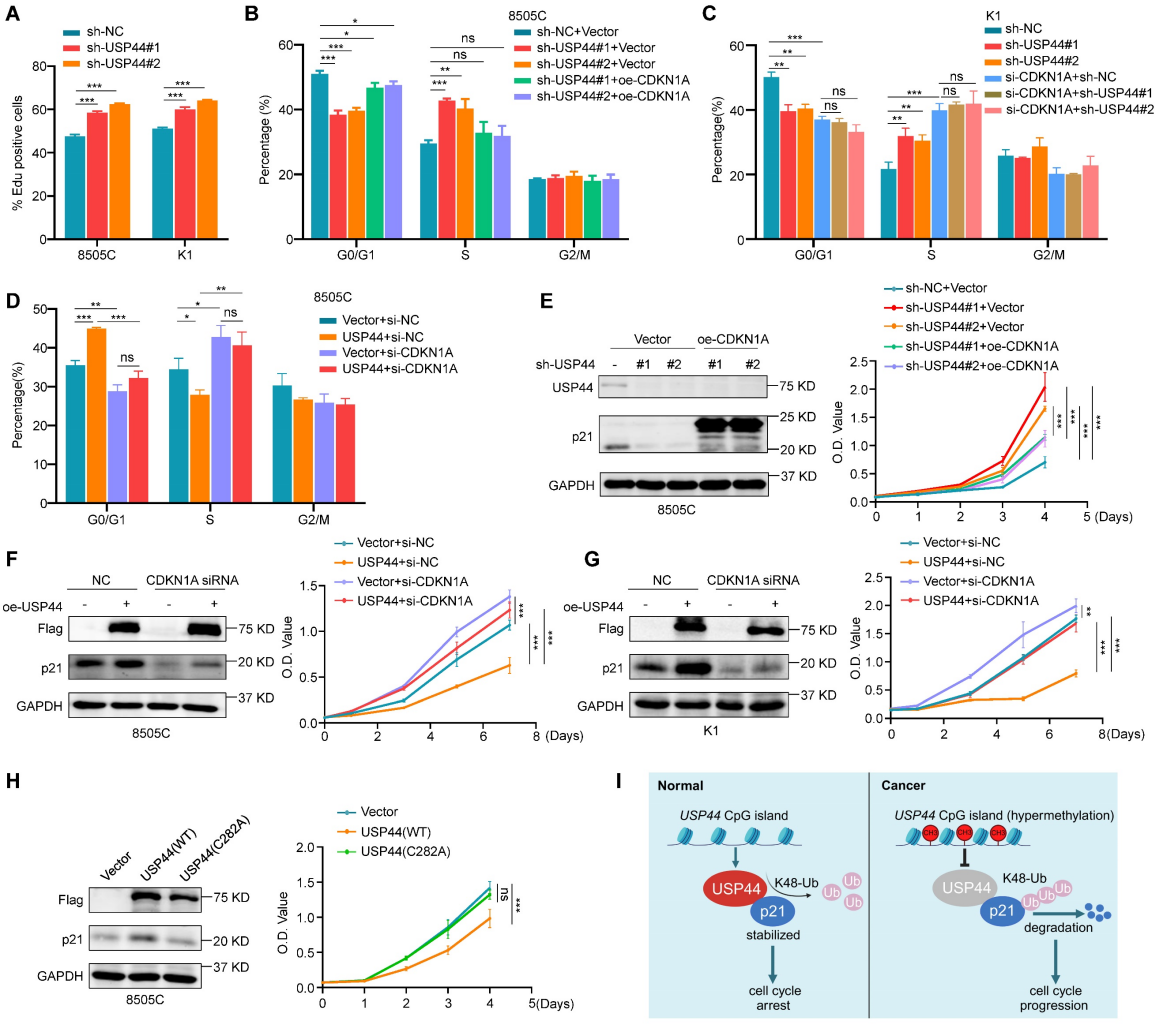
**USP44 regulates cell cycle progression in a p21-dependent manner. A** 8505C and K1 cells were synchronized through a double thymidine block followed by EdU incorporation assay, and the bar graph showing the proportion of EdU-positive cells. **B** Cell cycle distribution of USP44-knockdown 8505C cells and control cells was analyzed by flow cytometry in the presence or absence of ectopic expression of p21. **C** Cell cycle distribution of USP44-knockdown K1 cells and control cells was analyzed by flow cytometry in the presence or absence of siRNA-mediated p21 downregulation. **D** Cell cycle distribution of USP44-expressing 8505C cells and control cells was analyzed by flow cytometry in the presence or absence of siRNA-mediated p21 downregulation. **E** The effect of ectopic expression of p21 on the proliferation of USP44-knockdown 8505C cells was evaluated by MTT assay. Western blotting in the left panel shows the efficiency of USP44 knockdown and p21 overexpression. Right panel shows the effect of the above treatments on cell proliferation. **F** The effect of p21 knockdown on the proliferation of USP44-expressing 8505C cells and K1 cells (**G**) was evaluated by MTT assay. Western blotting in the left panel shows the efficiency of USP44 overexpression and p21 knockdown. Right panel shows the effect of the above treatments on cell proliferation. **H** The effects of ectopic expression of wild-type Flag-USP44 or Flag-USP44 mutant C282A on p21 expression (left panel) and cell proliferation (right panel) were evaluated by western blotting and MTT assays. **I** A simple model of the mechanism that USP44 interacts with and deubiquitinates p21 to inhibit cell cycle progression of thyroid cancer cells. However, promoter hypermethylation-mediated USP44 downregulation promotes thyroid tumorigenesis and progression by inducing ubiquitination and degradation of p21. GAPDH was used as a loading control for western blotting analysis. The data were presented as the mean ± SD from three independent experiments. *, *P* < 0.05; **,* P* < 0.01; ***, *P* < 0.001; ns, no significance.

Given that p21 contributes to cell cycle arrest, we propose that USP44 can potentially control the cell cycle transition from G1 to S phase. To further verify this hypothesis, we conducted EdU cell proliferation assays to evaluate the percentage of cells in the S phase under conditions with or without double-thymidine block and release. As expected, USP44 knockdown increased the proportion of EdU-positive cells in the S phase (Fig. 8A; Fig. S4A, B). We also ectopically expressed p21 in USP44-knockdown 8505C cells, showing the re-expression of p21 could alleviate the promoting effect of USP44 knockdown on cell cycle progression (Fig. 8B; Fig. S5A). Similarly, USP44 knockdown resulted in effects on the G1/S phase transition that were comparable to those observed with CDKN1A (gene encoding p21) knockdown. However, knocking down USP44 in CDKN1A-knockdown K1 cells failed to increase the proportion of S phage cells (Fig. 8C; Fig. S5B). Moreover, ectopic expression of USP44 in CDKN1A-knockdown cells did not decrease the proportion of S phase cells (Fig. 8D; Fig. S6A).

We also ectopically expressed p21 in USP44-knockdown 8505C cells (Fig. 8E, left panel) and knocked down p21 in USP44-overexpressing cells (Fig. 8F-G, left panel), and then performed MTT assays to evaluate their effect on cell proliferation. The results showed that the promoting effect of USP44 knockdown on cell proliferation could be abolished by ectopic expression of p21 (Fig. 8E, right panel). As expected, the inhibitory effect of USP44 overexpression on cell proliferation could be reversed by p21 knockdown (Fig. 8F-G, right panel). Meanwhile, we also ectopically expressed wild-type USP44 and USP44-C282A mutant in 8505C cells, and expectedly found that the former but not the latter upregulated the levels of p21 (Fig. 8H, left panel) and inhibited cell proliferation (Fig. 8H, right panel). Collectively, these data indicate that USP44 mediates G1/S transition and suppresses the growth of thyroid cancer cells by regulating p21expression. Since the expression of cell cycle-related proteins is contingent upon the cell cycle stage, we next determined whether the effect of USP44 on p21 was linked to a particular phase of cell cycle by using synchronized cells at various cell cycle stages. Strikingly, USP44 knockdown significantly decreased the levels of p21 across all cell cycle phases (Fig. S7A-B), indicating that USP44 stabilizes p21 in a cell-cycle-independent manner.

Combining the above findings, we propose a schematic model to elucidate the molecular mechanism by which USP44 exerts the tumor-suppressing function in thyroid cancer cells (Fig. 8I). Briefly, USP44 interacts with and stabilizes p21 proteins by removing its K48-linked polyubiquitin chains to suppress proteasome-mediated p21 degradation, thereby inducing cell cycle arrest. However, USP44 is downregulated or even inactivated by promoter hypermethylation in thyroid cancer, thus losing its tumor-suppressing function.

## Discussion

The pathogenesis of thyroid cancer involves a complex interplay of genetic and epigenetic events 35. In DTCs, the prevalent genetic alterations encompass mutations in *BRAF* and *RAS*, *RET* gene fusions and rearrangements, and *PIK3CA* mutations, which lead to constitutive activation of the MAPK/ERK or PI3K/AKT signaling pathways. Subsequent molecular events, such as *TP53* and *TERT* promoter mutations, further facilitate the dedifferentiation of DTC into PDTC and even ATC 36. However, epigenetic events also play a critical role in the development and progression of thyroid cancer, including DNA methylation, histone deacetylation, long non-coding RNAs and chromatin remodeling 37. Among them, aberrant DNA methylation serves as a major mechanism of the inactivation of tumor-suppressor genes, contributing to the pathogenesis and progression of thyroid cancer 34. In this study, we found that *USP44* was frequently downregulated by promoter methylation in thyroid cancers, as confirmed by MSP assays and demethylation treatment in a panel of thyroid cancer cell lines. Moreover, we also observed that *USP44* expression was negatively associated with *BRAF^V600E^* mutation, tumor stage, histological type, extrathyroidal extension and thyroid gland disorder history, and reduced *USP44* expression was positively correlated with poor clinical outcomes of patients. Importantly, *USP44* may also serve as a potential biomarker for the diagnosis of PTC.

*USP44*, belonging to the USP family, is implicated in regulating various physiological functions and pathological processes, including cell cycle regulation, chromosome segregation, immune response, stem cell differentiation, DNA damage response and tumor progression 8, 10, 11, 38-41. Previous studies have indicated that *USP44* functions as a tumor suppressor in hepatocellular carcinoma, pancreatic cancer, colorectal cancer, renal clear cell carcinoma and non‑small lung cancer 38, 40, 42-44. Notably, *Usp44* knockout animals exhibited an increased incidence of spontaneously arising tumors due to heightened chromosome segregation errors, further supporting its tumor-suppressing function 8. Nonetheless, the specific functions and underlying mechanisms of *USP44* in thyroid cancer remain to be comprehensively elucidated. In this study, we provided compelling evidence to support USP44 as a tumor suppressor gene in thyroid cancer. Specifically, USP44 inhibited the proliferation and colony formation abilities of thyroid cancer cells, reduced tumor growth in nude mice and transgenic mice and induced G1/S arrest. Notably, ATC cell lines usually exhibit greater malignancy than PTC cell lines in the tumorigenicity assays using nude mice, resulting in more pronounced tumor formation. Thus, we used 8505C cells exclusively to evaluate the effect of USP44 on tumorigenic potential in nude mice, which was one of the limitations of this study.

To explore the mechanism underlying the tumor-suppressing function of USP44 in thyroid cancer, we first identified USP44-interacted proteins by liquid chromatography tandem mass spectrometry (LC-MS/MS) combined with co-IP. Of them, cyclin-dependent kinase inhibitor p21 attracted our attention because it is a well-characterized downstream effector of p53, and functions as a tumor suppressor by inducing cell cycle arrest in tumorigenesis including thyroid cancer 19, 45. Besides, considering that the half-life of p21 proteins is relatively short, with its stability predominantly regulated by posttranslational modifications such as ubiquitination and phosphorylation 20, 39, we speculate that USP44 plays its tumor suppressor function in thyroid cancer cells by regulating the protein stability of p21 via deubiquitination. First, we confirmed the physical interaction between USP44 and p21 by co-IP, GST pull-down and immunofluorescence assays. Next, we demonstrated that knocking down USP44 in thyroid cancer cells reduced p21 protein levels, while ectopic expression of wild-type USP44 but not its enzymatically inactive mutant, led to an increase in p21 protein levels, indicating that USP44 regulates p21 protein expression dependent on its deubiquitinating enzyme activity. Given that p21 is a downstream target of p53, we attempt to rule out that the regulatory effect of USP44 on p21 is dependent on the p53 pathway. Our data showed that overexpression or knockdown of USP44 almost did not affect the protein expression of p53 and mRNA levels of *CDKN1A* in thyroid cancer cells, indicating that USP44 regulates p21 expression independently of p53. Next, we demonstrated that USP44 significantly increased the protein stability of p21 by inhibiting its proteasome degradation. Further studies showed that USP44 removed the K48-linked polyubiquitin chain of p21 through its deubiquitinase activity.

It is well-known that p21 acts as a key negative regulator of cell cycle progression 16 and operates as a tumor suppressor in nuclear localization 46. Based on these findings, we speculate that USP44 inhibits G1/S transition by stabilizing the expression of p21. Indeed, our findings showed that USP44 overexpression significantly inhibited the G1/S transition and cell proliferation of thyroid cancer cells, and vice versa. Meanwhile, we also demonstrated that the promoting effect of USP44 knockdown on cell cycle progression and cell proliferation could be attenuated by re-expression of p21, while p21 downregulation reversed the inhibitory effect of USP44 overexpression on cell cycle progression and cell proliferation, further supporting the above conclusion. In addition, our data also revealed that USP44 stabilized p21 proteins in a cell-cycle-independent manner.

In summary, our findings indicate that *USP44* is frequently downregulated due to promoter methylation in thyroid cancer and is strongly associated with a poor patient prognosis. Additionally, we demonstrate that USP44 functions as a tumor suppressor by a series of *in vitro* functional experiments as well as xenograft and transgenic mouse models. Mechanistically, USP44 interacts with p21 and removes its K48-linked polyubiquitination chains, thereby protecting p21 from proteasome-mediated degradation and maintaining its protein stability. However, promoter hypermethylation-mediated *USP44* downregulation promotes thyroid tumorigenesis and progression.

## Supplementary Material

Supplementary figures and tables.

## Abbreviations

TCGA: The Cancer Genome Atlas
GEO: Gene Expression Omnibus
PTC: Papillary thyroid cancer
FTC: Follicular thyroid cancer
MTC: Medullary thyroid cancer
PDTC: Poorly differentiated thyroid cancer
ATC: Anaplastic thyroid cancer
DUBs: Deubiquitylating enzymes
USP44: Ubiquitin-specific protease 44
PFI: progress-free interval
CHX: cycloheximide
