# Supplementary material for: USP44 inactivation accelerates the progression of thyroid cancer by inducing ubiquitylation and degradation of p21
Source: Int J Biol Sci. 2024 Sep 23;20(13):5223–38. doi: 10.7150/ijbs.99817 (PMC11489182; doi:10.7150/ijbs.99817)
Supplement: Supplementary file 1 — Supplementary figures and tables. [file ijbsv20p5223s1.pdf]

Supplementary Data

Table S1. The primers used in this study for various PCR assays

Genes	Forward primer (5'-3')	Reverse primer (5'-3')
**qRT-PCR assay**		
USP44	ACAACTTATGATATGCCACC	GATTTCCTCAAAGCCAAC
CDKN1A	TGTCCGTCAGAACCCATGC	AAAGTCGAAGTTCCATCGCTC
β-actin	CCTTGCACATGCCGGAG	GCACAGAGCCTCGCCTT
**MSP assay**		
USP44-M	TAGAAATTTTAGGAAGGATTAACGAC	CTATAATACCGTCGAAACACGAA
USP44-U	TAGAAATTTTAGGAAGGATTAATGATGT	CCTATAATACCATCAAAACACAAA
**RT-PCR**		
Braf	TGAGTATTTTTGTGGCAACTGC	CTCTGCTGGGAAAGCGGC
Tpo-Cre	AGGTGTAGAGAAGGCACTTAGC	CTAATCGCCATCTTCCAGCAGG

Table S2. siRNAs and shRNAs used in this study

siRNAs/shRNAs	Sense (5'-3')	Antisense (5'-3')
si-USP44#1	GAACAUGGUUUGAACAAUC	GAUUGUUCAAACCAUGUUC
si-USP44#2	GAAUUGGAGUAUCAAGUUA	UAACUUGAUACUCCAAUUC
si-NC	UUCUCCGAACGUGUCACGU	ACGUGACACGUUCGGAGAA
si-CDKN1A	CUUCGACUUUGUCACCGAG	CUCGGUGACAAAGUCGAAG
sh-NC (for sh-USP44)	GATCCGTTCTCCGAACGTGTCACGT AATTCAAGAGATTACGTGACACGTT CGGAGAA	
sh-USP44#1	GCACAGGAGAAGGATACTAATCTC GAGATTAGTATCCTTCTCCTGTGC	
sh-USP44#2	CCTGTTGCATTGGAGGTGAATCTCG AGATTCACCTCCAATGCAACAGG	

Table S3. Antibodies used in this study

Antibodies	Catalog	Source
anti-USP44	ab205032	abcam
anti-USP44	A08401-2	BOSTER
anti-USP44	TA801913	ORIGENE
anti-p21	2947S	Cell Signaling Technologies
anti-p21	ab188224	abcam
anti-Flag	F3165	Sigma
anti-Myc	2276S	Cell Signaling Technologies
anti-ubiquitin	ab-134953	abcam
anti-HA	3724S	Cell Signaling Technologies
anti-p53	sc-126	Santa Cruz
anti-GAPDH	AP0063	Bioworld
anti-Ki67	ab15580	abcam
anti-His tag	230001	ZENBIO
anti-GST tag	300195	ZENBIO

Table S4. Demographic and clinicopathological features of PTC patients with high and low USP44 expression (data from TCGA database).

**Characteristics**	**Low *USP44* expression**	**High *USP44* expression**	***P* value**
n	256	256	
Pathologic T stage, n (%)			< 0.001
T1	53 (10.4%)	90 (17.6%)	
T2	79 (15.5%)	90 (17.6%)	
T3	104 (20.4%)	71 (13.9%)	
T4	19 (3.7%)	4 (0.8%)	
Pathologic N stage, n (%)			0.002
N0	98 (21.2%)	131 (28.4%)	
N1	133 (28.8%)	100 (21.6%)	
Pathologic M stage, n (%)			0.545
M0	145 (49.2%)	141 (47.8%)	
M1	6 (2%)	3 (1%)	
Pathologic stage, n (%)			< 0.001
Stage I	128 (25.1%)	160 (31.4%)	
Stage II	21 (4.1%)	31 (6.1%)	
Stage III	63 (12.4%)	50 (9.8%)	
Stage IV	44 (8.6%)	13 (2.5%)	
Gender, n (%)			0.487
Female	183 (35.7%)	190 (37.1%)	
Male	73 (14.3%)	66 (12.9%)	
Race, n (%)			0.091
Asian	21 (5%)	30 (7.2%)	
Black or African American	12 (2.9%)	15 (3.6%)	
White	189 (45.4%)	149 (35.8%)	
Age, n (%)			0.027

Characteristics	Low *USP4*4 expression	High *USP44* expression	*P* value
<= 45	109 (21.3%)	134 (26.2%)	
> 45	147 (28.7%)	122 (23.8%)	
Histological type, n (%)			< 0.001
Classical	194 (37.9%)	172 (33.6%)	
Follicular	29 (5.7%)	72 (14.1%)	
Tall Cell	28 (5.5%)	8 (1.6%)	
Other	5 (1%)	4 (0.8%)	
Residual tumor, n (%)			0.132
R0	195 (43.3%)	197 (43.8%)	
R1&R2	35 (7.8%)	23 (5.1%)	
Extrathyroidal extension, n (%)			< 0.001
No	145 (29.4%)	195 (39.5%)	
Yes	104 (21.1%)	50 (10.1%)	
Primary neoplasm focus type, n (%)			0.051
Unifocal	147 (29.3%)	122 (24.3%)	
Multifocal	107 (21.3%)	126 (25.1%)	
Neoplasm location, n (%)			0.823
Left lobe	89 (17.6%)	89 (17.6%)	
Right lobe	109 (21.5%)	109 (21.5%)	
Bilateral	42 (8.3%)	46 (9.1%)	
Isthmus	13 (2.6%)	9 (1.8%)	
Thyroid gland disorder history, n (%)			0.008
Normal	162 (35.7%)	124 (27.3%)	
Lymphocytic Thyroiditis	31 (6.8%)	43 (9.5%)	
Nodular Hyperplasia	25 (5.5%)	43 (9.5%)	
Other, specify	14 (3.1%)	12 (2.6%)	
OS event, n (%)			0.310

**Characteristics**	**Low *USP44* expression**	**High *USP44* expression**	***P* value**
Alive	246 (48%)	250 (48.8%)	
Dead	10 (2%)	6 (1.2%)	

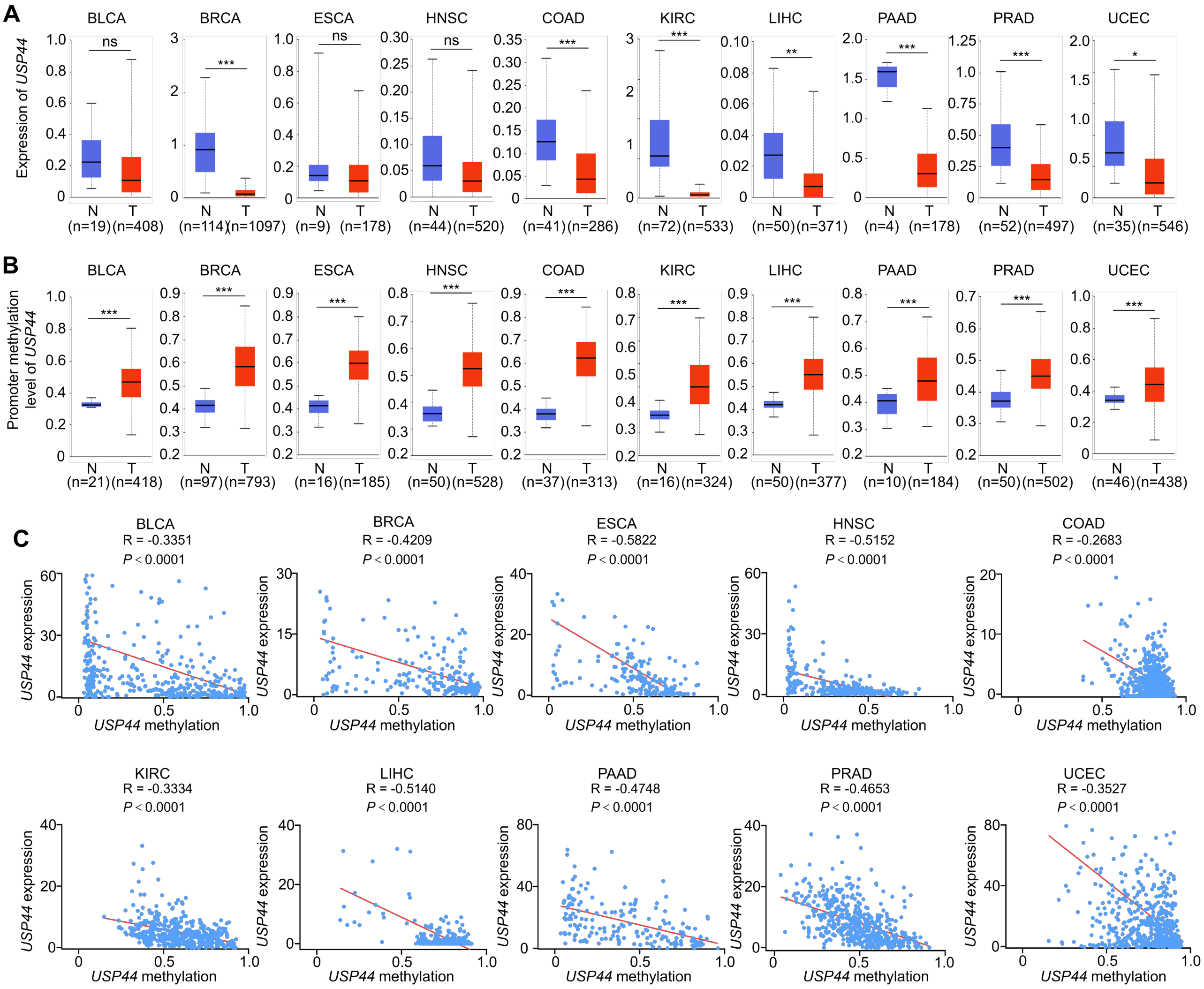

Fig. S1 Frequent downregulation of USP44 by promoter methylation in different types of human cancers**.** *USP44* mRNA levels (**A**) and methylation levels (**B**) in different tumors and normal tissues using UALCAN tool based on The Cancer Genome Atlas (TCGA) database. **C** The correlation analysis of USP44 methylation and mRNA expression levels in different tumors from TCGA database. The data were analyzed using the spearman correlation. BLCA bladder urothelial carcinoma, BRCA breast invasive carcinoma, ESCA esophageal carcinoma, HNSC head and neck squamous cell carcinoma, COAD colon adenocarcinoma, KIRC kidney renal clear cell carcinoma, LIHC liver hepatocellular carcinoma, PAAD pancreatic adenocarcinoma, PRAD prostate adenocarcinoma, UCEC uterine corpus endometrial carcinoma. *, $P < 0.05$; **, $P < 0.01$; ***, $P < 0.001$; ns, no significance.

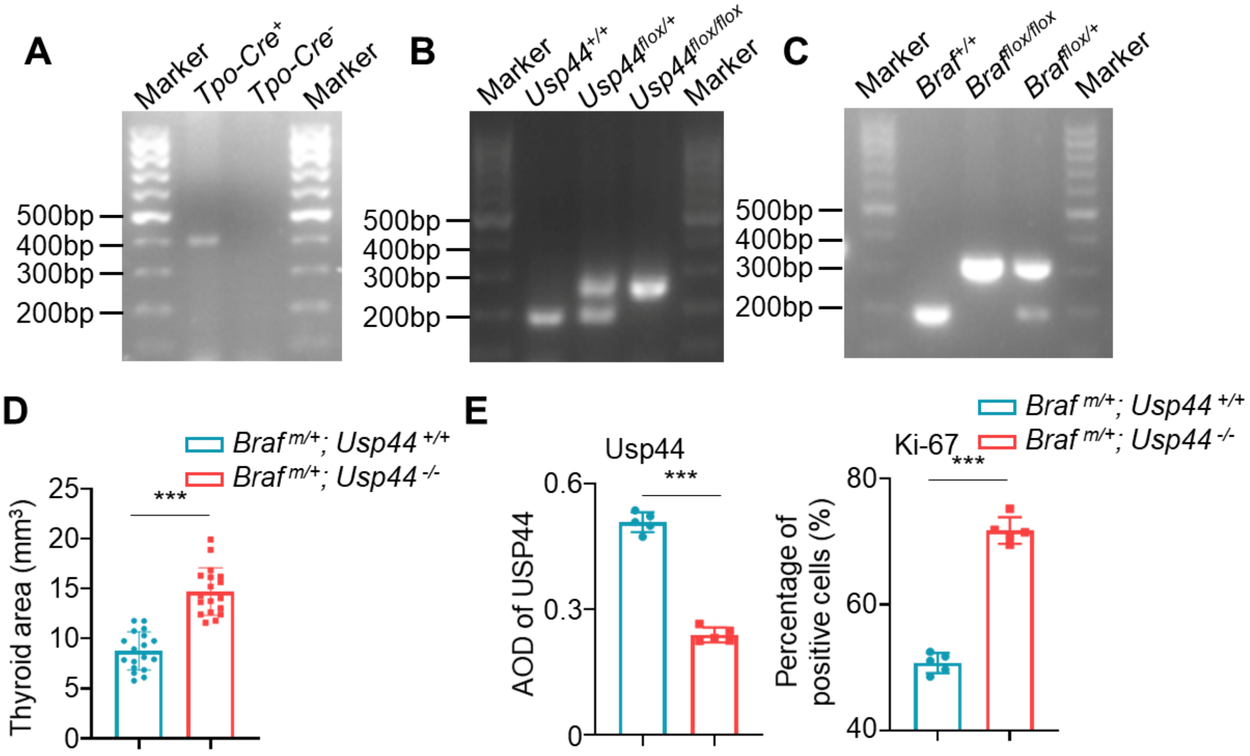

Fig. S2 USP44 knockout promotes tumor growth in transgenic mice. **A-C** Genotyping of genetically engineered mice. Marker, DNA marker. **D** Statistical analysis of ultrasound images of thyroid in transgenic mice with indicated genotypes. The data were shown as the mean ± SD. **E** The corresponding statistical plots for Usp44 and Ki-67 in tumor tissues from transgenic mice with different genotypes. The data were shown as the mean ± SD. ***, $P < 0.001$.

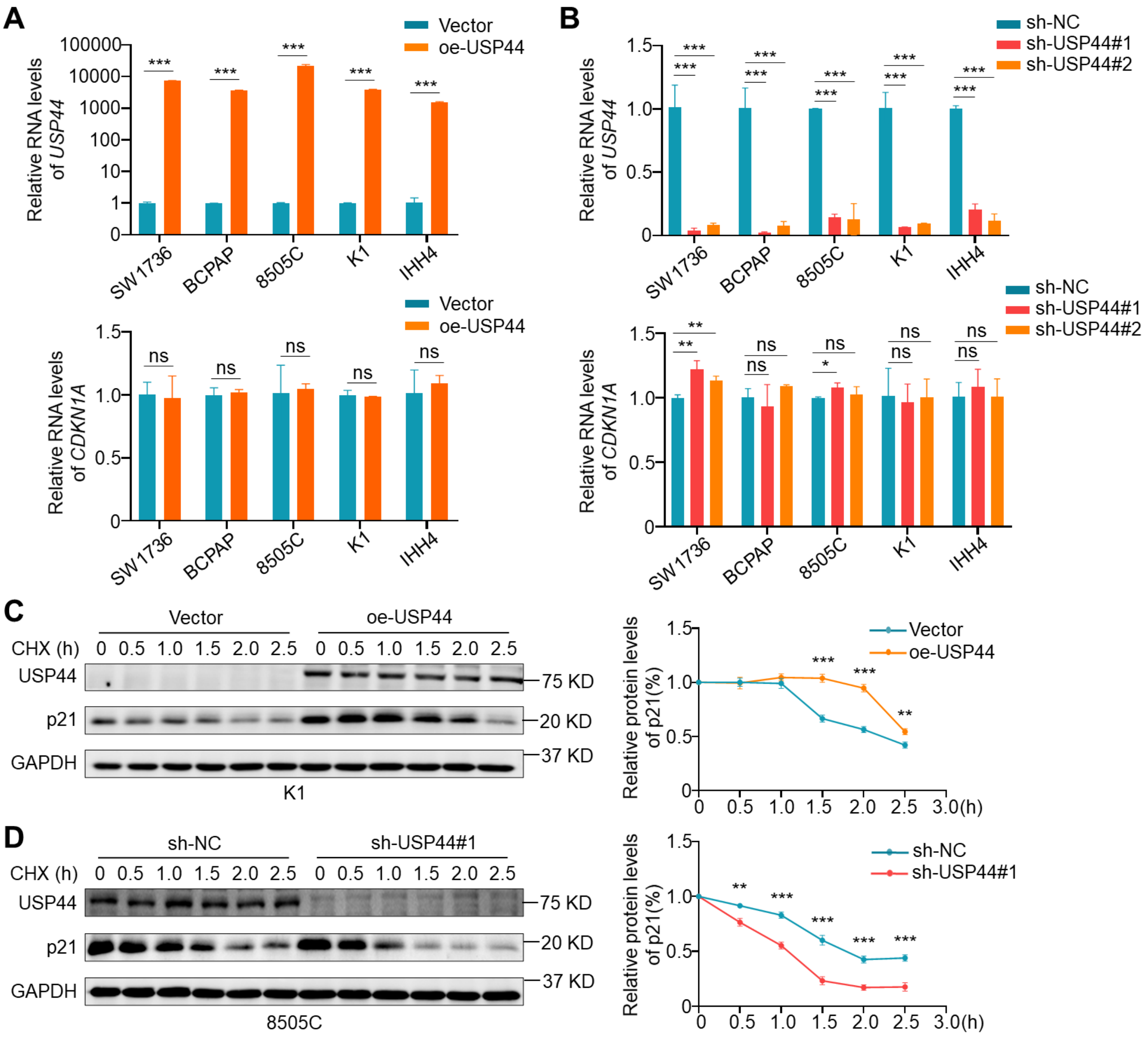

Fig. S3 USP44 increases the protein stability of p21. qRT-PCR assays were performed to evaluate the effects of overexpression (**A**) or knockdown (**B**) USP44 on mRNA expression levels of *CDKN1A* in the indicated thyroid cancer cells. β-Actin was used as the normalization control. USP44-overexpressing K1 cells (**C**) and USP44-knockdown 8505C cells (**D**) as well as their control cells were treated with 200 μg/mL CHX and collected at the indicated time points for western blotting analysis (left panels). Quantification of p21 protein levels relative to GAPDH was shown in the right panels. GAPDH was used as a loading control. The data were presented as the mean ± SD from three independent experiments. *, $P < 0.05$; **, $P < 0.01$; ***, $P < 0.001$; ns, no significance.

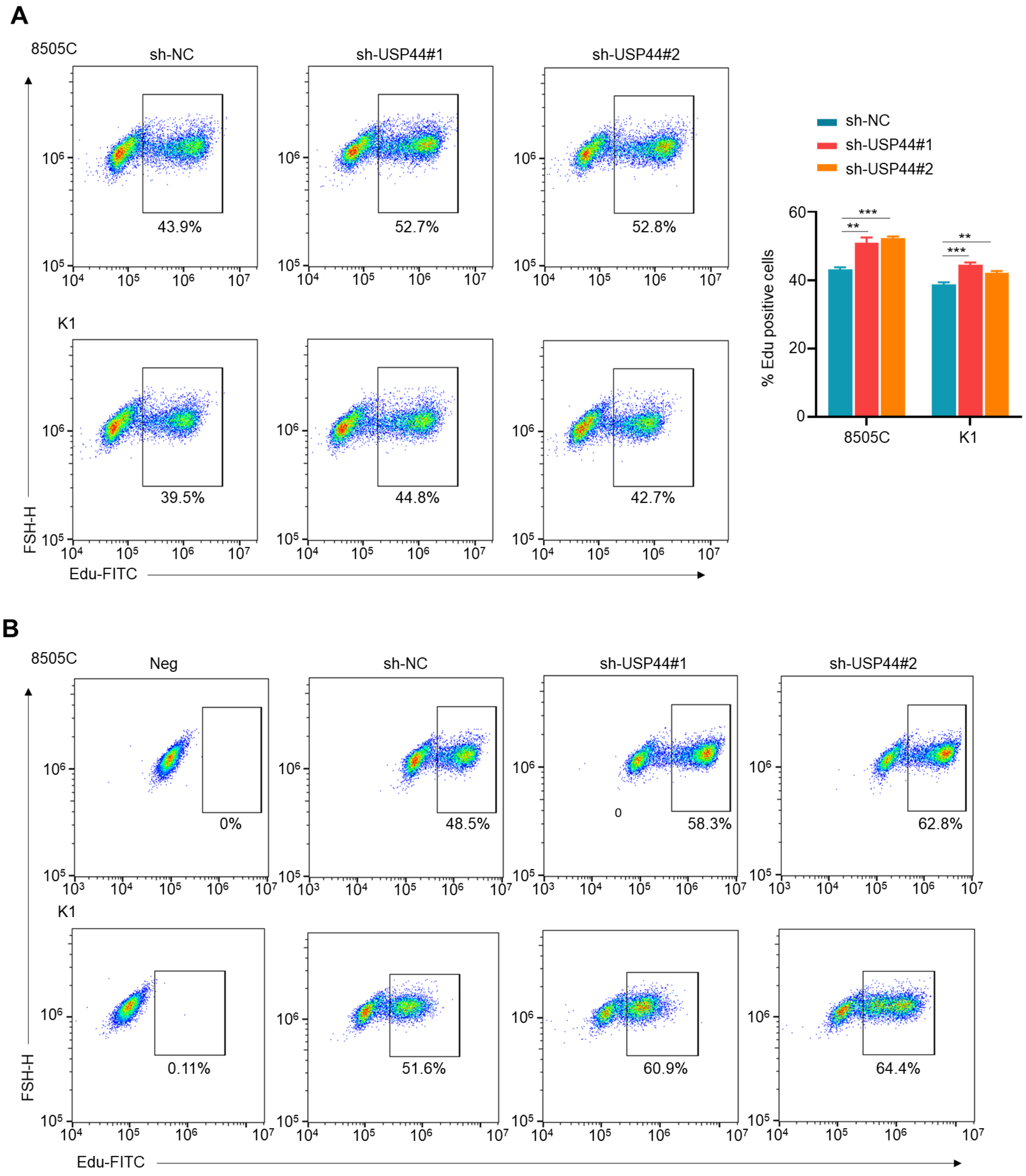

Fig. S4 USP44 regulates the G_1/S phase transition in thyroid cancer cells. **A** USP44-knockdown 8505C and K1 cells were cooperated with EdU for 150 min before harvesting and then analyzed using flow cytometry. Representative images were shown in the left panels, and the quantitative analysis results were shown in the right panel. **B** USP44-knockdown 8505C and K1 cells were synchronized at G_1/S phase border using a double

thymidine block followed by EdU incorporation assay. The data were presented as the mean ± SD from three independent experiments. **, $P < 0.01$; ***, $P < 0.001$.

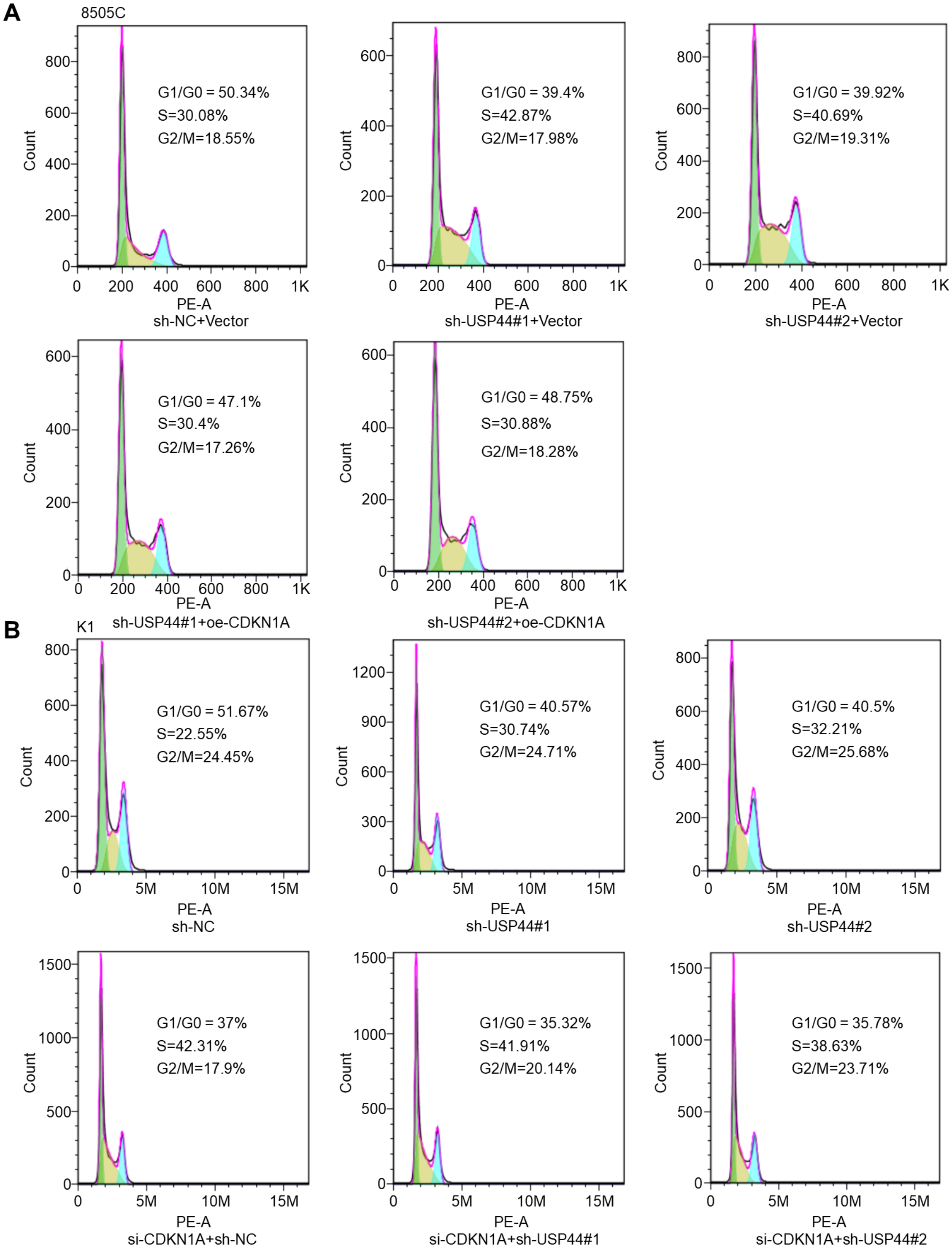

Fig. S5 USP44 regulates the G_1/S transition in a p21-dependent manner. **A** USP44-knockdown 8505C cells in the presence or absence of ectopic expression of p21 were stained with propidium iodide and analyzed by flow cytometry. **B** USP44-knockdown K1 cells in the presence or absence of siRNA-mediated p21 knockdown were stained with propidium iodide and analyzed by flow cytometry.

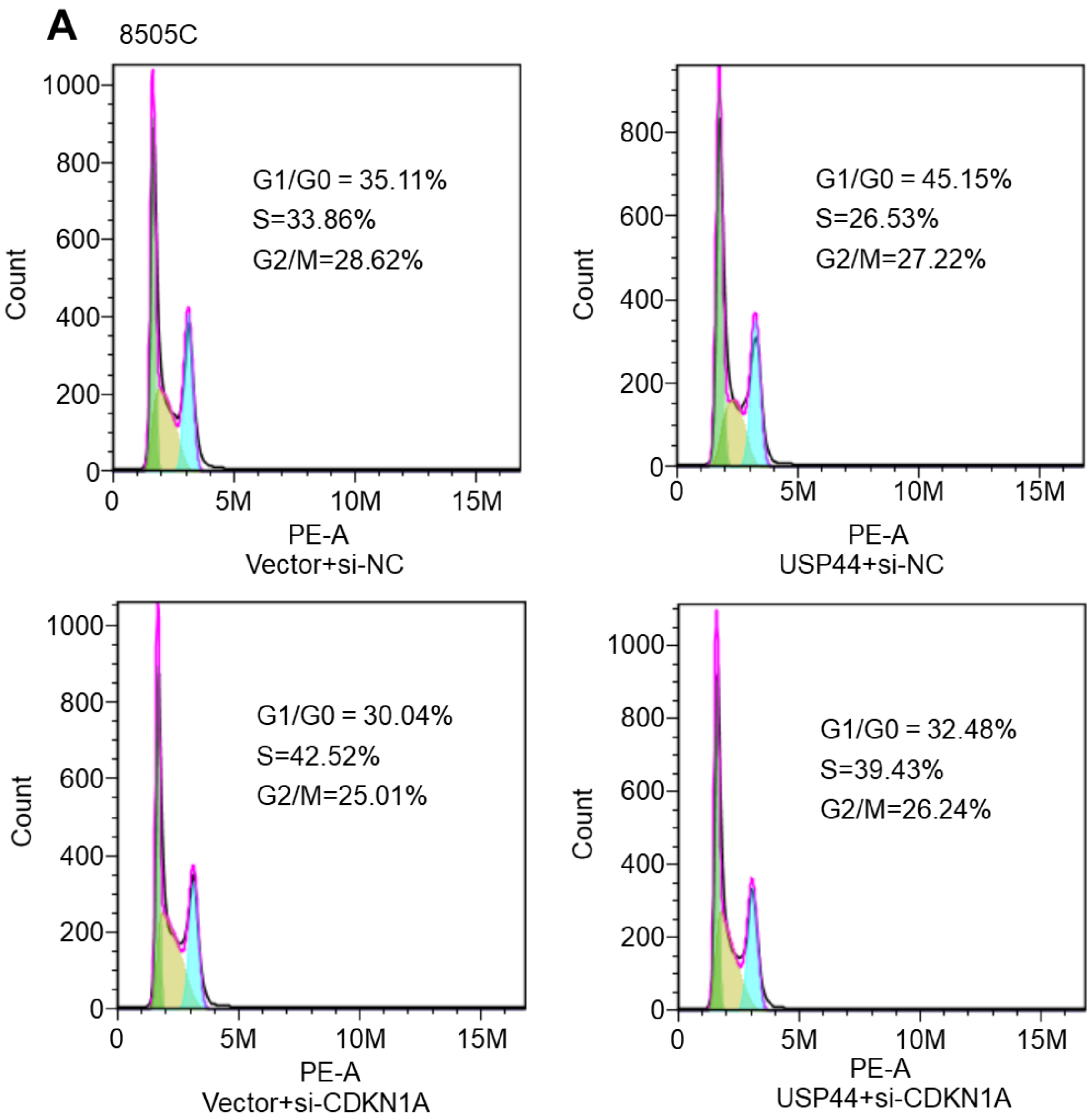

Fig. S6 USP44 regulates cell cycle progression in a p21-dependent manner. **A** Cell cycle distribution of USP44-expressing 8505C cells was analyzed by flow cytometry in the presence or absence of siRNA-mediated p21 downregulation.

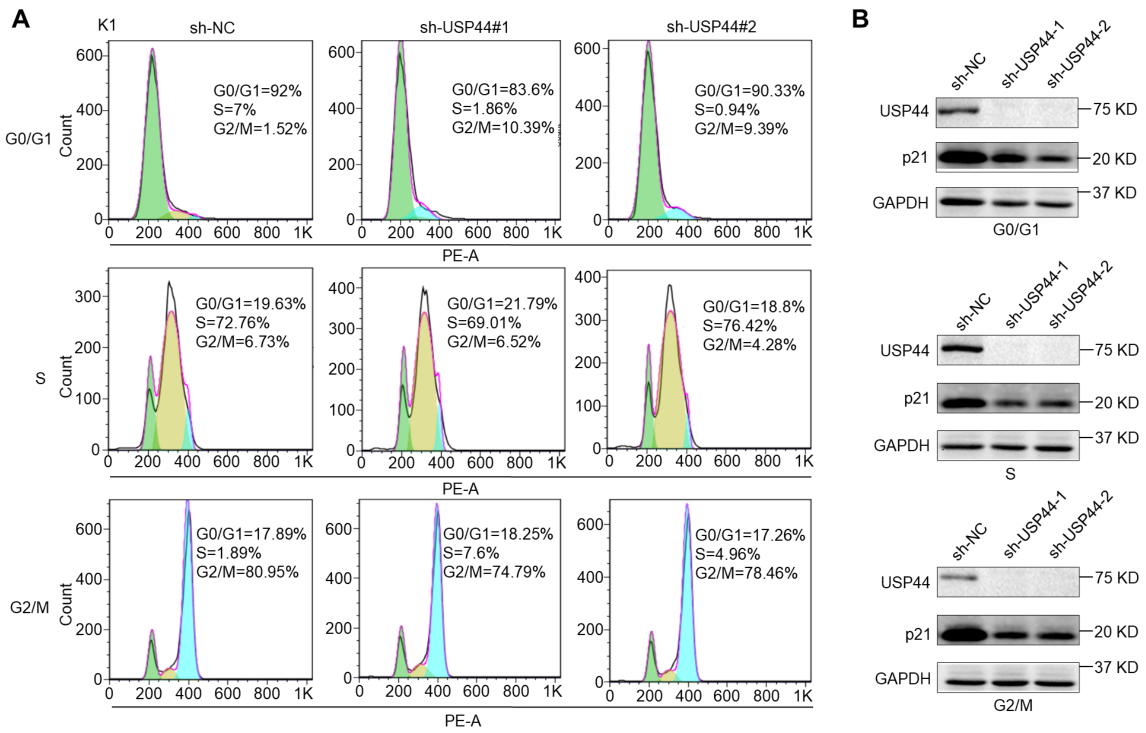

Fig. S7 USP44 stabilizes p21 in a cell cycle–independent manner. USP44-knockdown K1 cells were synchronized using a double-thymidine block and released at the indicated phase. One part of treated cells was then subjected to flow cytometry analysis (**A**) and another part of treated cells were collected for western blotting analysis (**B**). GAPDH was used as a loading control.
